# Marriage and physical capability at mid to later life in England and the USA

**DOI:** 10.1371/journal.pone.0209388

**Published:** 2019-01-23

**Authors:** Natasha Wood, Anne McMunn, Elizabeth Webb, Mai Stafford

**Affiliations:** 1 CLOSER, UCL Institute of Education, London, United Kingdom; 2 Department of Epidemiology and Public Health, University College London, London, United Kingdom; 3 The Health Foundation, London, United Kingdom; University of Colorado Denver, UNITED STATES

## Abstract

**Background:**

Married people have lower rates of mortality and report better physical and mental health at older ages, compared to their unmarried counterparts. However, there is limited evidence on the association between marriage and physical capability, the ability to carry out the tasks of daily living, which is predictive of future mortality and social care use. We investigate the association between marital status and physical capability at mid to later life in England and the United States.

**Methods:**

We examine the association between marriage and physical capability at mid to later life in England and the USA using two performance-based measures of physical capability: grip strength and walking speed. Multiple linear regression was carried out on Wave 4 (2008) of the English Longitudinal Study of Ageing (ELSA) and Waves 8 and 9 (2006 and 2008) of the US Health and Retirement Study (HRS).

**Results:**

In age adjusted models married men and women had better physical capability than their unmarried counterparts. Much of the marriage advantage was explained by the greater wealth of married people. However, remarried men were found to have stronger grip strength and widowed and never married men had a slower walking speed than men in their first marriage, which was not explained by wealth, demographic and socioeconomic characteristics, health behaviours, chronic disease or depressive symptoms. There were no differences in the association between England and the USA.

**Conclusions:**

Marriage may be an important factor in maintaining physical capability in both England and the USA, particularly because of the greater wealth which married people have accrued by the time they reach older ages. The grip strength advantage for remarried men may be due to unobserved selective factors into remarriage.

## Introduction

Research shows that those who are married have better physical and psychological health and greater longevity than their unmarried counterparts [1] [2] [3], as well as better health than those who are in unmarried cohabiting relationships [4]. There is mixed evidence on whether men’s health benefits more from marriage relative to women’s [5] [3]. Different explanations have been proposed for the association, including that marriage protects health through increased economic resources, improved health behaviours and the provision of social support (1). Alternatively, unmarried people’s experiences of transitions out of marriage may be deleterious to health because of the accompanying stress, emotional upheaval, subsequent lapse in health behaviours and loss of economic resources [6]. The third explanation is that marriage is *s*elective of those who have better physical and mental health [7] [8] [9] [10] and greater economic resources [11] in the first instance.

Despite the health advantages associated with marriage, the past 40 to 50 years have witnessed a decline in marriage, an increase in the prevalence of divorce and a rise in unmarried cohabitation in both England and the USA [12]. Consequently, there are now more people entering mid to later life unmarried which, given the strong association between marriage and health and coupled with the ageing population, could result in an increase in numbers with poorer health at older ages.

Whilst there is much evidence showing the association between marriage and physical and mental health, little is known about the association between marriage and physical capability. Physical capability is the capacity to undertake the physical tasks of daily living [13] and is a key indicator of healthy ageing, not specific to a particular disease or condition [14] [15]. Physical capability is predictive of subsequent disability [16] and mortality [17], is associated with physical health [18] and has shown to be predictive of social care use, including entry into long-term care [19] and admission to hospital [20].

Existing evidence on marriage and physical capability has largely used self-reported measures of activities of daily living (ADL) or mobility limitations, and found that those who are married report fewer limitations than their unmarried counterparts [21] [22]. The few studies that have used the performance-based measures of physical capability, which measure strength, balance, coordination and flexibility, have also found that married people have better physical capability than those who are unmarried [23] [24]. There is also some evidence of gender differences in the association with never married men having relatively poorer physical capability than never married women [21] [23].

The majority of studies which have investigated marriage and physical capability have also treated those who are married as a homogenous group, not distinguishing between first marriage and subsequent marriages. These two groups of people have differing relationship histories, with those who are remarried having experienced a transition out of marriage, which could modify any association with physical capability. At present it is unknown whether those who are remarried have similar levels of physical capability to those who have remained in their first marriage, although there is some evidence that those who are in a subsequent marriage have a higher number of activity limitations than those in their first marriage [25].

The association between marriage and physical capability may be modified by national context. England and the United States of America (USA) are two countries which are useful to compare as although they hold many similarities there are some key differences between them which may alter any association between marriage and physical capability. Firstly, there are differences in marriage and divorce patterns, the USA has higher marriage and divorce rates than England [26], therefore divorce may be a more normative experience for people in the USA and consequently may have a weaker association with physical capability. Alternatively, evidence shows that income levels drop post-divorce, particularly for women, who not only have lower incomes from paid work than men in the first instance, but are also more likely to be the main provider of childcare which may limit their participation in paid work [27] [28]. Consequently, those who are divorced may be more reliant upon welfare provision. Welfare provision in England is more generous than in the USA, particularly through universal free health care. Therefore we may expect to see a stronger association between marriage and physical capability in the USA than in England, particularly for women. There is also evidence of physical health differentials between England and the USA at older ages [29], as well as differences in levels of physical capability at older ages, with those in the USA having poorer physical capability than their counterparts in England [30] [31]. All of these differences could translate into differing associations between marriage and physical capability, which could further our understanding of the underlying mechanisms. To the best of our knowledge there is no research which has investigated the differences in the association between marriage and performance-based physical capability in England and the USA.

In this article nationally representative data from England and the USA are used to investigate: whether the performance-based measures of physical capability vary by marital status, differentiating between those who are in their first marriage and those in a subsequent marriage; whether there are differences by gender; and whether the associations vary between England and the USA. The contribution of socioeconomic, behavioural and health factors to these associations were also tested.

## Methods

### Data

Data were drawn from the English Longitudinal Study of Ageing (ELSA) and the US Health and Retirement Study (HRS). These are nationally representative longitudinal studies of people aged 50 years and older in England [32], and of people aged 51 years and older in the USA [33]. Both surveys are part of a wider group of international harmonised longitudinal studies on ageing and share many of the same measures, which makes them ideal for cross-national comparative research. Ethical approval for ELSA was obtained from the UK National Research and Ethics Committee and for HRS from the University of Michigan’s Institutional Review Board.

HRS administers the performance based measures on alternating halves of its sample at each wave; therefore, in order to obtain a sample with a complete set of outcome measures, we pooled the two sample halves from two successive waves: Wave 8 (2006) and Wave 9 (2008). All the other HRS variables included in this analysis were taken from the year in which the participant’s performance-based tests were collected. We used Waves 8 and 9 of HRS as these were the first waves that the physical performance tests had been administered. In order to ensure that the measures used in ELSA were collected in a comparable time frame to those in HRS, data from Wave 4 (2008) was used.

### Physical capability

Two measures of physical capability were utilised: grip strength and walking speed; both of which have been widely used in research based in England and the USA [15] [34] [35] [36]. Grip strength was measured on both studies using a Smedley Dynamometer. The highest grip strength measurement out of the first two tests on each hand was used and was adjusted for height in metres. We adjusted for height in metres because of the well-documented direct positive correlation between height and grip strength [37] [38] [39]. Walking speed was measured by respondents walking 2.44 metres (8 feet) in ELSA and 2.50 metres (8.2 feet) in HRS, whilst being timed. The test is performed twice and the mean time in metres per second from the two tests was used. The test is performed on all those aged 60 years and older in ELSA and aged 65 years and older in HRS, so for comparability the analysis of walking speed was restricted to those aged 65 years and older in ELSA. Those who were unable to do the tests for health reasons (grip strength: ELSA *n* = 86 out of a sample of 7,478; HRS *n* = 349 out of a sample of 12,750; walking speed: ELSA *n* = 223 out of sample of 3,645; HRS *n* = 538 out of a sample of 8,337) were given an age and sex adjusted mean value representative of the bottom quintile, as it would be expected that those who were unable to perform the tests due to health reasons would have poor physical capability. This approach has been used previously for the performance-based measures [40] and more information is provided in Tables A and B in S1 File. We ran a sensitivity analysis to investigate the robustness of this approach and whether excluding those who were unable to do the tests from the analysis, or giving these individuals either their gender age specific mean score, or gender age specific lowest score gave divergent results. The analysis (provided in S1 File) showed that the results were similar overall to those using the gender age specific bottom quintile score.

### Marital status

Our exposure of interest was current legal marital status. The current marital status measure distinguished between those in a first marriage and those in a subsequent marriage and was categorised into first marriage, remarried, divorced / separated, widowed and never married. In ELSA the marital status measure collected at each wave differentiated between those in a first marriage and those in a remarriage, however in HRS this was not the case and instead the RAND derived variable [41], which identified how many times a respondent had been married at each wave, was used to create a remarried category. Those who were in a same-sex civil partnership (ELSA only n = 17) were assigned to either the first marriage or remarried category, dependent on their prior marital status. Those who were unmarried but cohabiting were assigned their legal marital status (ELSA n = 392 and HRS n = 422).

### Covariates

The analysis adjusted for a number of covariates to help explain any association between marital status and physical capability, comprising demographic, socioeconomic, health behaviour and physical and mental health measures.

Demographic covariates include age, sex, ethnicity, and work and parental status. Age was included as a non-linear term as physical capability declines faster at older ages [42]. In ELSA ethnicity was dichotomised into white and non-white, due to ELSA having insufficiently large sample sizes of England’s ethnic minority groups to categorise ethnicity in a more detailed way. In HRS ethnicity was categorised into white / Hispanic / black / other to reflect the ethnic composition of the USA.

Education and wealth have been used to measure socioeconomic position. Education was measured using the number of years of full time education and was divided into three categories: low (0–11 years of schooling, O-level equivalent, in ELSA; 0–12 years, high school or less, in HRS), medium (12–13 years, A-level equivalent, in ELSA; 13–15 years, more than high school but not a college graduate, in HRS) and high (14+ years, higher education qualification, in ELSA; 16+ years, college or more, in HRS). Education was categorised in this way to be broadly equivalent to the International Standard Classification of Education 2011 and the same categorisation has been employed in prior comparative research using ELSA and HRS [29] [43] [44].

Wealth was used rather than income as it has shown to be a more appropriate measure of socioeconomic position at older ages (32). Both HRS and ELSA contain detailed questions on income, assets and debt, which make it possible to derive accurate measures of wealth. Total wealth was used, which is the sum of savings, investments, physical wealth and housing wealth after financial and mortgage debt have been subtracted. Wealth was measured at the couple level and was categorised into quintiles from low to high.

A number of health behaviours including physical activity, smoking status and BMI were included in the analysis. Physical activity, including any physical activity from both leisure activities and paid work, was self-reported and categorised into sedentary, low, moderate, and high. Smoking status was categorised into never smoked, ever smoked and current smokers. Objectively measured height and weight were used to derive body mass index (BMI), which was categorised according to the World Health Organisation guidelines: 0–24.9 kg/m^2^ (underweight to normal weight); 25–29.9 kg/m^2^ (overweight); 30+ kg/m^2^ (obese). The underweight and normal weight categories were combined as only relatively small numbers of participants were underweight (ELSA underweight *n* = 68; HRS underweight *n* = 180). BMI was categorised, rather than treated as continuous, since the association between physical capability and BMI was not linear.

Three measures of physical and mental health were included: self-rated health; number of doctor diagnosed health conditions and depressive symptoms. The measure of self-rated health was identical on both surveys and was collapsed into three categories: excellent to very good; good; and fair to poor. Reported number of doctor-diagnosed chronic health conditions comprised hypertension, diabetes, cancer, chronic lung disease, heart disease, stroke and arthritis and was categorised into 0 conditions, 1 condition, 2 conditions, and 3 or more conditions. Depressive symptoms were captured by the 8-item version of the Centre for Epidemiologic Studies Depression Scale (CES-D), which has been dichotomised into <3 and ≥3 depressive symptoms, as 3 or more symptoms are indicative of clinical depression [45].

### Analytic sample and statistical analysis

**Fig 1:**
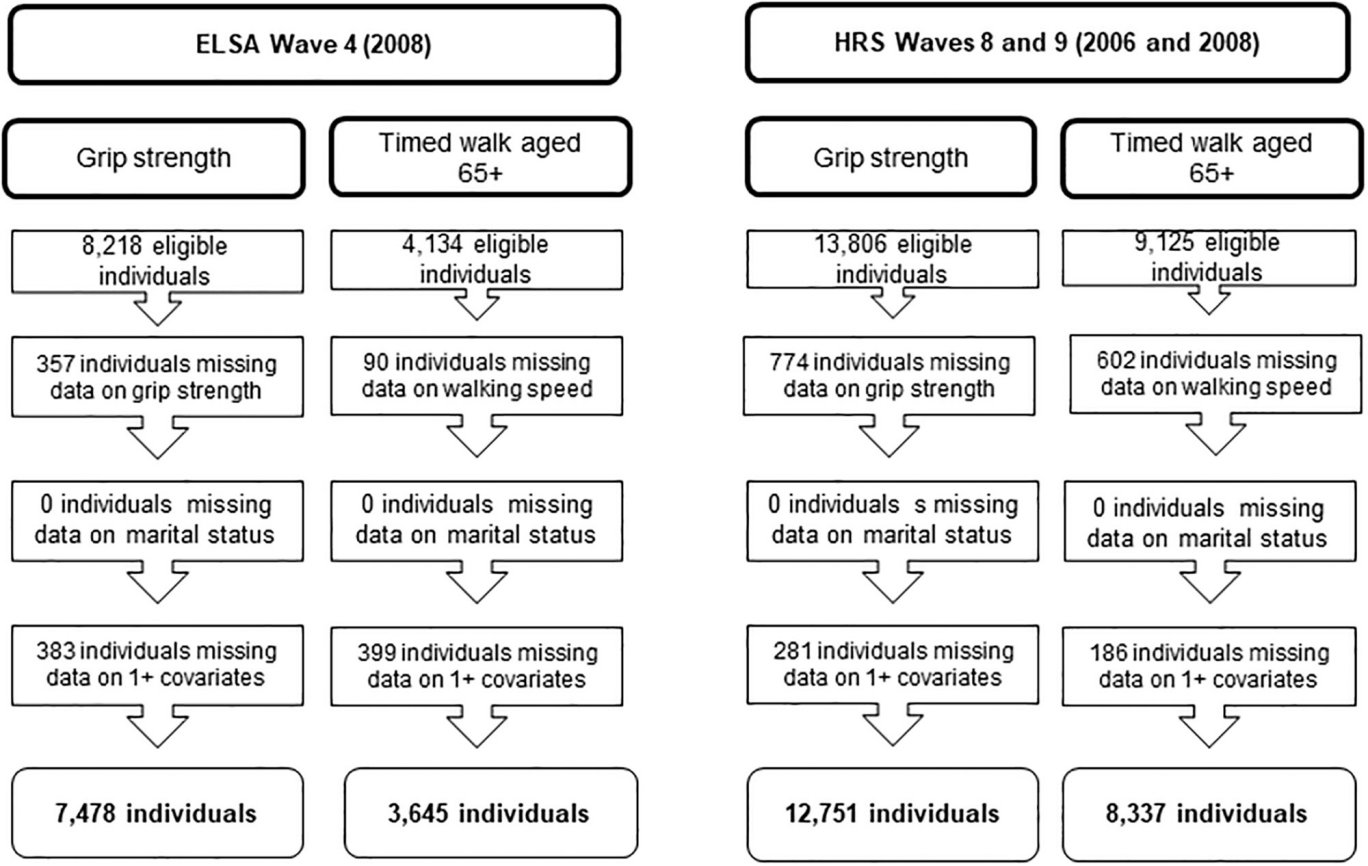
Breakdown of the analytical samples for grip strength and walking speed in ELSA and HRS.

The final analytic samples in ELSA and HRS comprised only cases with complete data. There were different analytic samples for the analysis of grip strength and walking speed. This was largely because the walking speed analysis was restricted to those aged 65 years and over as 65 was the minimum age walking speed was measured in HRS. Details of the analytic samples in ELSA and HRS are shown in Fig 1.

Overall there were few marital status differences between the complete sample and the analytic sample, but more detail on any potential bias introduced due to differences between the analytic sample and the cases which were omitted is provided in Tables A and B in S2 File.

Linear regression was used to estimate mean differences in grip strength and walking speed according to marital status. Linear regression was used as both grip strength and walking speed were normally distributed. The base model included age and demographic characteristics (model 1). To explore socioeconomic, behavioural and health variables that might lie on the explanatory pathways linking marital status to physical capability, we additionally adjusted for education and wealth (model 2), and physical activity, smoking status, BMI, self-rated health, chronic health conditions and depressive symptoms (model 3).

The analysis was carried out separately in ELSA and HRS and stratified by gender. Both HRS and ELSA samples contain couples and stratification by gender avoids problems due to clustering in physical capability at the household level. We tested for marital status by gender interactions separately on each survey and then combined the data for both surveys and tested for marital status by country interactions for men and for women.

## Results

Table 1 shows the characteristics of the ELSA and HRS analytic samples. On the measures of physical capability individuals in HRS had stronger mean grip strength than individuals in ELSA, whilst individuals in ELSA had faster mean walking speed than those in HRS. Men in both surveys had stronger mean grip strength and faster mean walking speed than women. A higher percentage in ELSA remained in their first marriage than in HRS and a higher percentage in HRS were remarried or divorced. There were higher percentages whom were never married in ELSA than in HRS. Overall, the HRS sample was also older, comprised more women, more highly educated, more likely to be parents, and more likely to have chronic health conditions than the ELSA sample.

**Table 1:** Characteristics of the ELSA and HRS samples.

	ELSA		HRS	
	*Men (N = 3*,*391)* ^a^	*Women (N = 4*,*129)* ^a^	*Men (N = 5*,*512)* ^a^	*Women (N = 7*,*591)* ^a^
	*Mean (SE) or %*	*Mean (SE) or %*	*Mean (SE) or %*	*Mean (SE) or %*
**Mean highest grip strength (kg /m)**^b^	**22.47(0.08)**	**14.81 (0.06)**	**23.15(0.06)**	**15.08 (0.05)**
**Mean walking speed (m/s)**^b^	**0.857 (0.007)**	**0.782 (0.006)**	**0.779 (0.004)**	**0.699 (0.004)**
**Marital status**				
First marriage	**61.0**	**47.8**	**51.7**	**37.0**
Remarried	**14.5**	**11.9**	**25.2**	**15.7**
Divorced / separated	**10.1**	**14.3**	**11.1**	**14.5**
Widowed	**8.0**	**20.9**	**8.9**	**29.8**
Never married	**6.5**	**5.1**	**3.1**	**3.0**
**Age**				
50–59	**28.6**	**28.7**	**20.1**	**19.3**
60–69	**38.8**	**37.0**	**32.8**	**33.6**
70–79	**24.2**	**24.6**	**31.9**	**30.5**
80+	**8.4**	**9.8**	**15.3**	**16.6**
**Ethnicity**				
White	**97.1**	**97.6**	**78.8**	**74.8**
Non-white (ELSA) / Hispanic (HRS)	2.9	2.4	8.2	8.7
Black (HRS only)	-	-	11.6	15.1
Other (HRS only)	-	-	1.4	1.4
**Work status**				
Working	**40.6**	**31.4**	**35.5**	**28.1**
Not working	**59.4**	**68.6**	**64.5**	**71.9**
**Parental status**				
Has children	**84.2**	**85.0**	**94.1**	**94.3**
No children	**15.8**	**15.0**	**5.9**	**5.7**
**Education**				
Low	**45.3**	**45.0**	**51.7**	**59.6**
Medium	**35.2**	**39.7**	**20.2**	**22.1**
High	**19.5**	**15.3**	**28.1**	**18.3**
**Wealth (country-specific quintile)**				
1st—low wealth	**14.4**	**17.3**	**15.4**	**21.2**
2^nd^	17.9	19.6	18.2	21.2
3^rd^	20.1	20.4	21.2	19.7
4^th^	23.2	**20.9**	21.8	**19.2**
5^th^ high wealth	24.4	**21.8**	23.4	**18.6**
**Smoking status**				
Never smoked	32.4	**46.2**	32.1	**52.2**
Former smoker	**54.9**	**40.3**	**52.3**	**34.0**
Current smoker	**12.7**	**13.5**	**15.6**	**13.8**
**Physical activity**				
Sedentary	4.8	**5.3**	6.0	**5.2**
Low	**17.3**	**27.2**	**21.0**	**31.4**
Moderate	**52.9**	**49.9**	**40.2**	**39.9**
High	**24.9**	**17.6**	**32.8**	**23.4**
**Body Mass Index**				
Underweight to normal weight (≤24)	**21.8**	31.0	**26.9**	33.6
Overweight BMI (25–29)	**49.2**	**35.8**	**41.2**	**31.8**
Obese BMI (≥30)	**29.0**	**33.2**	**31.9**	**34.6**
**Self-rated health**				
Excellent / v. good	44.2	41.8	41.3	40.1
Good	31.5	33.0	31.3	31.7
Fair / poor	**24.3**	**25.2**	**27.4**	**28.2**
**Chronic health conditions**				
0 reported conditions	**32.8**	**28.3**	**14.2**	**11.3**
Reported 1 condition	**31.3**	**32.7**	**24.6**	**24.8**
Reported 2 conditions	**21.4**	**22.6**	**26.6**	**29.9**
Reported 3+ conditions	**14.5**	**16.3**	**34.6**	**34.1**
**CES-D**				
CES-D<3	**85.3**	75.0	**84.0**	76.3
CES-D≥3	14.7	25.0	16.0	23.7

Significant country differences, p<0.05, between ELSA and HRS men and ELSA and HRS women are highlighted in bold.

^a^ Totals comprise cases which were included in the grip strength or timed walk analytic samples.

^b^ Mean grip strength and walking speed are age adjusted.

### Grip strength

Tables 2 and 3 summarise the regression analysis of marital status on grip strength for men and women in ELSA and HRS. Tables showing the effect sizes for all the covariates in the models are given in S3 File.

**Table 2:** Regression coefficients for grip strength (in kg / height in m) for men in ELSA and HRS.

	Model 1: age and demographics		Model 2: age, demographics + SEP		Model 3: age demographics + SEP + health behaviours and health	
	Coeff	95% CI	Coeff	95% CI	Coeff	95% CI
***ELSA***						
**Marital status (first marriage ref category)**						
Remarried	**0.61**	**(0.15, 1.07)**	**0.76**	**(0.30, 1.22)**	**0.72**	**(0.27, 1.16)**
Divorced / separated	-0.10	(-0.63, 0.44)	0.48	(-0.07, 1.03)	0.52	(-0.02, 1.07)
Widowed	**-0.73**	**(-1.36, -0.11)**	-0.43	(-1.05, 0.19)	-0.40	(-1.01, 0.21)
Never married	-0.61	(-1.38, 0.15)	-0.26	(-1.02, 0.51)	-0.13	(-0.88, 0.61)
***HRS***						
**Marital status (first marriage ref category)**						
Remarried	0.22	(-0.08, 0.51)	**0.30**	**(0.01, 0.59)**	**0.31**	**(0.03, 0.59)**
Divorced / separated	**-0.62**	**(-1.02, -0.21)**	-0.20	(-0.61, 0.21)	-0.05	(-0.45, 0.35)
Widowed	**-0.80**	**(-1.26, -0.35)**	**-0.53**	**(-0.99, -0.07)**	-0.41	(-0.85, 0.03)
Never married	**-1.45**	**(-2.28, -0.62)**	**-1.07**	**(-1.89, -0.24)**	**-0.97**	**(-1.77, -0.18)**

Results p<0.05 are shown in bold.

Model 1: Age and demographics (ethnicity, work status and parental status)

Model 2: Age + demographic and socioeconomic measures (education and wealth)

Model 3: Age + demographic and socioeconomic measures + health behaviours (smoking status, physical activity and BMI) + physical health and mental health (self-rated health, chronic health conditions and depressive symptoms)

**Table 3:** Regression coefficients for grip strength (in kg / height in m) for women in ELSA and HRS.

	Model 1: age and demographics		Model 2: age, demographics + SEP		Model 3: age demographics + SEP + health behaviours and health	
	Coeff	95% CI	Coeff	95% CI	Coeff	95% CI
***ELSA***						
**Marital status (first marriage ref category)**						
Remarried	0.14	(-0.21, 0.48)	0.29	(-0.05, 0.63)	**0.36**	**(0.03, 0.69)**
Divorced / separated	**-0.42**	**(-0.74, -0.10)**	-0.06	(-0.39, 0.27)	0.04	(-0.28, 0.37)
Widowed	-0.31	(-0.61, 0.00)	-0.08	(-0.39, 0.23)	0.01	(-0.29, 0.32)
Never married	-0.48	(-1.02, 0.07)	-0.31	(-0.86, 0.24)	-0.27	(-0.79, 0.26)
***HRS***						
**Marital status (first marriage ref category)**						
Remarried	-0.11	(-0.34, 0.12)	-0.04	(-0.27, 0.19)	-0.01	(-0.24, 0.21)
Divorced / separated	**-0.29**	**(-0.54, -0.05)**	0.03	(-0.22, 0.29)	0.04	(-0.21, 0.29)
Widowed	**-0.49**	**(-0.70, -0.29)**	**-0.24**	**(-0.46, -0.03)**	-0.20	(-0.41, 0.01)
Never married	-0.02	(-0.54, 0.49)	0.30	(-0.22, 0.82)	0.24	(-0.26, 0.74)

Results p<0.05 are shown in bold.

Model 1: Age and demographics (ethnicity, work status and parental status)

Model 2: Age + demographic and socioeconomic measures (education and wealth)

Model 3: Age + demographic and socioeconomic measures + health behaviours (smoking status, physical activity and BMI) + physical health and mental health (self-rated health, chronic health conditions and depressive symptoms)

#### Men

After adjusting for age and demographic measures (model 1), widowed and never married men in ELSA had a weaker grip strength than men in their first marriage (0.73 kg/m and 0.61 kg/m weaker, respectively). In HRS all groups of unmarried men had a weaker grip strength than men in their first marriage. In both samples much of the association was attenuated once additionally adjusting for the socioeconomic measures (model 2), and it was largely wealth which explained the weaker grip strength among widowed and never married men and among divorced men in HRS. Remarried men in both ELSA and HRS had stronger grip strength than men in their first marriage (0.61 kg/m stronger in ELSA and 0.22 kg/m stronger in HRS) when adjusting for the demographic measures (model 1) and further adjusting for the socioeconomic measures (model 2) and health behaviours, physical health and mental health (model 3) did not attenuate the association.

We tested for interactions by country and there was no moderation in the association by country (results not shown).

#### Women

Table 3 shows the results for women. In model 1 (adjusted for the demographic measures) women in ELSA who were divorced or never married had a weaker grip strength than women in their first marriage (0.42 kg/m and 0.48 kg/m weaker, respectively) and this was attenuated once adjusting for the socioeconomic measures (model 2), with wealth accounting for most of the attenuation. In model 1 (adjusted for demographic measures) HRS divorced and widowed women had weaker grip strength than those in their first marriage (0.29 kg/m and 0.49 kg/m weaker respectively); for divorced women the association was attenuated when adjusting for the socioeconomic measures (model 2). The socioeconomic measures partly attenuated widowed women’s weaker grip strength, but the association was fully attenuated on the introduction of the health behaviours and the physical and mental health measures (model 3). Similar patterns were observed in ELSA and HRS and there was no moderation by country among women.

Among women in both ELSA and HRS there was not as much variation in grip strength among the different marital statuses as among men. There was some moderation in the association by gender between marital status and grip strength for those who were widowed, never married and remarried. Remarried men had relatively stronger grip strength than remarried women, whilst widowed and never married women had relatively stronger grip strength than their male counterparts.

### Walking speed

The same models were run for marital status and walking speed.

#### Men

Table 4 summarises the association between marital status and walking speed for men. When adjusting for the demographic measures (in model 1) all unmarried men had a slower walking speed than men who were in their first marriage, in both surveys. The slower walking speed among divorced men was explained by the socioeconomic measures (model 2), primarily wealth. Among widowed men in both samples and never married men in ELSA, the socioeconomic measures (model 2) only partly attenuated their slower walking speed and adjustment for health behaviours, physical health and mental health did little to attenuate this further (model 3). There was no evidence that the association between marital status and walking speed was different for men in ELSA to those in HRS.

**Table 4:** Regression coefficients for walking speed (in metres per second) among men aged 65 years and older, in ELSA and HRS.

	Model 1: age and demographics		Model 2: age, demographics + SEP		Model 3: age demographics + SEP + health behaviours and health	
	Coeff	95% CI	Coeff	95% CI	Coeff	95% CI
***ELSA***						
**Marital status (first marriage ref category)**						
Remarried	-0.002	(-0.041, 0.037)	0.011	(-0.026, 0.048)	0.014	(-0.020, 0.048)
Divorced / separated	**-0.086**	**(-0.138, -0.034)**	-0.033	(-0.083, 0.018)	-0.015	(-0.061, 0.032)
Widowed	**-0.080**	**(-0.120, -0.041)**	**-0.046**	**(-0.085, -0.008)**	**-0.042**	**(-0.077, -0.006)**
Never married	**-0.113**	**(-0.187, -0.039)**	**-0.080**	**(-0.151, -0.010)**	**-0.082**	**(-0.147, -0.017)**
***HRS***						
**Marital status (first marriage ref category)**						
Remarried	0.004	(-0.016, 0.024)	0.012	(-0.008, 0.032)	0.016	(-0.003, 0.035)
Divorced / separated	**-0.034**	**(-0.064, -0.003)**	-0.001	(-0.032, 0.029)	-0.002	(-0.031, 0.027)
Widowed	**-0.068**	**(-0.094, -0.041)**	**-0.043**	**(-0.070, -0.017)**	**-0.037**	**(-0.062, -0.011)**
Never married	-0.035	(-0.101, 0.032)	-0.010	(-0.075, 0.056)	-0.021	(-0.083, 0.041)

Results p<0.05 are shown in bold.

Model 1: Age and demographics (ethnicity, work status and parental status)

Model 2: Age + demographic and socioeconomic measures (education and wealth)

Model 3: Age + demographic and socioeconomic measures + health behaviours (smoking status, physical activity and BMI) + physical health and mental health (self-rated health, chronic health conditions and depressive symptoms)

#### Women

Among women the demographic adjusted model (model 1) showed that unmarried women in both ELSA and HRS had a slower walking speed than women in their first marriage (Table 5), and the addition of the socioeconomic measures (model 2), primarily wealth, attenuated this association. There was no evidence that the association between walking speed and marital status was different for women in ELSA than in HRS, and there was no evidence that the association was moderated by gender in either ELSA or HRS.

**Table 5:** Regression coefficients for walking speed (in metres per second) among women aged 65 years and older, in ELSA and HRS.

	Model 1: age and demographics		Model 2: age, demographics + SEP		Model 3: age demographics + SEP + health behaviours and health	
	Coeff	95% CI	Coeff	95% CI	Coeff	95% CI
***ELSA***						
**Marital status (first marriage ref category)**						
Remarried	-0.032	(-0.075, 0.012)	-0.009	(-0.051, 0.033)	0.020	(-0.017, 0.056)
Divorced / separated	**-0.057**	**(-0.096, -0.018)**	0.004	(-0.035, 0.042)	-0.002	(-0.036, 0.032)
Widowed	**-0.057**	**(-0.085, -0.030)**	-0.013	(-0.040, 0.014)	0.000	(-0.024, 0.024)
Never married	**-0.075**	**(-0.140, -0.011)**	-0.046	(-0.108, 0.016)	-0.033	(-0.087, 0.022)
***HRS***						
**Marital status (first marriage ref category)**						
Remarried	0.000	(-0.023, 0.023)	0.013	(-0.009, 0.035)	0.015	(-0.006, 0.036)
Divorced / separated	**-0.046**	**(-0.070, -0.023)**	-0.001	(-0.025, 0.023)	0.001	(-0.022, 0.023)
Widowed	**-0.051**	**(-0.068, -0.034)**	-0.014	(-0.031, 0.003)	-0.008	(-0.024, 0.007)
Never married	-0.043	(-0.095, 0.008)	-0.015	(-0.065, 0.035)	-0.028	(-0.074, 0.019)

Results p<0.05 are shown in bold.

Model 1: Age and demographics (ethnicity, work status and parental status)

Model 2: Age + demographic and socioeconomic measures (education and wealth)

Model 3: Age + demographic and socioeconomic measures + health behaviours (smoking status, physical activity and BMI) + physical health and mental health (self-rated health, chronic health conditions and depressive symptoms)

## Discussion

Using nationally representative data from two surveys of older people in England and the USA, an association was found between marriage and physical capability at mid to later life, with those who were unmarried displaying poorer physical capability than their counterparts who had remained in their first marriage. Our findings reinforce those from two existing studies, which also investigated the relationship between marital status and the performance-based measures of physical capability [23] [24], as we too found that never married men and widowed men and women had poorer physical capability than their married counterparts. Our findings also echo the findings from other research which have used the self-reported measures of physical capability [21] [22]. Much of the association was explained by the greater wealth of married people. Previous research has shown a strong association between marriage and wealth [46] [47] and between wealth and physical capability [48] [49], and this research shows that the greater economic resources associated with marriage are important for physical capability.

There was found to be some moderation in the association by gender, but only for the measure of grip strength. Being widowed or never married was associated with relatively weaker grip strength for men than for women, whilst remarriage for men was associated with relatively stronger grip strength than it was for women. Other studies have also found gender differences in the association, particularly among those who are never married [23]. However, our study extends on previous research by showing that there are differences in physical capability between those in a first marriage and those who are remarried.

These gender differences and the relative advantage of remarried men compared to men in their first marriage in the measure of grip strength could possibly be explained by gender specific selective factors into marriage, for instance men who are more muscular and stronger may be more likely to be selected into marriage in the first instance and then back into marriage after a marital transition. There were no such gender differences in the association between marital status and walking speed, which could be because walking speed is not so reliant upon muscle mass as grip strength [13].

The relative physical capability advantage of remarried men compared to men who have remained in their first marriage is in contrast to previous evidence [25]. The differences in findings could be due to the different measure of physical capability that were utilised, as the previous study used self-reported mobility to measure physical capability. More evidence is needed on remarriage and physical capability to determine the association.

Widowed and never married men had slower walking speeds than men in their first marriage, an association which remained after adjusting for all covariates. Prior research has also found widowed and never married men to have particularly poor physical capability compared to their married counterparts, but the association has yet to be fully explained [50] [51] [23]. It is possible that in this analysis we have not captured all the explanatory pathways linking marriage to physical capability. In particular, married men most commonly nominate their spouse or partner as their closest person providing social support [52] [53] and social support has been linked to physical capability [54] [55], although the evidence is inconsistent [56]. Consideration of social support was beyond the scope of the current study, but it could be important to consider in future work aiming to understand the link between marriage and physical capability.

There was consistency in the association of marital status with physical capability between England and the USA, which shows that the association is robust and not moderated by national context in this instance. This could be because England and the USA have similar cultures and social attitudes and further research may show differences between countries that have very different attitudes and social norms surrounding marriage.

There are a number of strengths and limitations to this analysis. A key strength is that it used two large comparable nationally representative datasets to investigate marriage and physical capability in an international context and sheds some light on this little researched area. The study also used the performance-based measures of physical capability, which are more accurate for international research as they are less prone to distortion by cultural and educational differences associated with the self-reported measures [34]. The analysis was carried out only on cases with complete data, which may have resulted in the analytical sample being biased. Where there were differences with the full sample, the unmarried participants with complete data had better physical capability than those who were excluded from the analyses. Thus we may have underestimated the physical capability disadvantage of being unmarried (see S2 File).

We were also limited by the lack of ethnic diversity within the ELSA sample and consequently we could not investigate ethnic differences in the association between marriage and physical capability (although we did adjust for ethnicity in the analysis). We note that marriage is experienced differently amongst various ethnic groups [57] and that further research looking into associations between marriage and physical capability within ethnic groups would be of value.

Given the association between marital status and physical capability, more people entering older ages never married, or having experienced a transition out of marriage could potentially mean more people experiencing poorer physical capability at older ages. The importance of wealth in explaining much of the poorer physical capability among older unmarried people suggests that increases in access to economic resources available to unmarried people may help to maintain physical capability and independent living at older ages.

## Supporting information

S1 File Unable to do tests due to health reasons. (DOCX)

S2 File Missing data analysis. (DOCX)

S3 File Complete models. (DOCX)
