# Supplementary material for: Marriage and physical capability at mid to later life in England and the USA
Source: PLoS One. 2019 Jan 23;14(1):e0209388. doi: 10.1371/journal.pone.0209388 (PMC6343866; doi:10.1371/journal.pone.0209388)
Supplement: S1 File — (DOCX) [file pone.0209388.s001.docx]

## S1: Unable to do the physical capability tests due to health reasons

### Analysis of who was unable to do the tests due to health reasons

S1A Table: Calculated grip strength values given to those who were unable to do the grip strength test due to health reasons

|  | ELSA | | | | HRS | | | |
| --- | --- | --- | --- | --- | --- | --- | --- | --- |
|  | **Men** | | **Women** | | **Men** | | **Women** | |
| Age | **Value (kg/m)** | **N** | **Value (kg/m)** | **N** | **Value (kg/m)** | **N** | **Value (kg/m)** | **N** |
| 50-59 | 18.17 | 6 | 11.26 | 11 | 19.86 | 13 | 12.61 | 49 |
| 60-69 | 16.86 | 6 | 10.67 | 18 | 17.37 | 23 | 11.37 | 88 |
| 70-79 | 14.59 | 15 | 8.59 | 14 | 15.42 | 18 | 9.77 | 84 |
| 80+ | 11.50 | 5 | 6.60 | 11 | 12.15 | 13 | 7.72 | 61 |
| *Total (N)* | ***32*** | | ***54*** | | ***67*** | | ***282*** | |

S1B Table: Calculated walking speed values given to those who were unable to do the walking speed test due to health reasons

|  | ELSA | | | | HRS | | | |
| --- | --- | --- | --- | --- | --- | --- | --- | --- |
|  | **Men** | | **Women** | | **Men** | | **Women** | |
|  | **Value**  **(m/s)** | **N** | **Value**  **(m/s)** | **N** | **Value**  **(m/s)** | **N** | **Value**  **(m/s)** | **N** |
| 60-69 | 0.610 | 17 | 0.554 | 23 | 0.518 | 39 | 0.465 | 66 |
| 70-79 | 0.490 | 32 | 0.427 | 74 | 0.468 | 74 | 0.389 | 132 |
| 80+ | 0.350 | 25 | 0.294 | 52 | 0.368 | 80 | 0.283 | 147 |
| *Total* | ***74*** | | ***149*** | | ***193*** | | ***345*** | |

Logistic regression analysis was carried out to investigate whether there were differentials by marital status in the odds of being unable to participate in the tests due to health reasons, adjusted for age. Table S1C and Table S1D show that whilst in ELSA there was no association between marital status and being unable to complete the grip strength measurement for health reasons, in HRS there was an association and men who were remarried were less likely than men in their first marriage to be unable to do the grip strength test due to health reasons, whilst divorced men and widowed women were more likely to be unable to do the test due to health reasons than those in their first marriage. For the measure of walking speed in ELSA there was only an association between marital status and walking speed among women, and women who were divorced were more likely to be unable to do the walking speed test for health reasons than women in their first marriage. In HRS a similar association were seen for the measure of walking speed as what was seen for the measure of grip strength and among men those who were remarried were less likely than those in their first marriage to be unable to do the test due to health reasons, whilst divorced men and widowed women were more likely than those in their first marriage to be unable to do the test. Removing individuals from the analysis who are unable to participate in the physical capability measures would result in an underestimation of the variation in physical capability by marital status for those who are unmarried largely in HRS, but also in ELSA.

S1C Table: Logistic regression coefficients showing the likelihood of unable to do the grip strength test due to health reasons by marital status adjusted for age

|  | ELSA | | | | HRS | | | |
| --- | --- | --- | --- | --- | --- | --- | --- | --- |
|  | **Men** | | **Women** | | **Men** | | **Women** | |
|  | **Coef** | **95% CI** | **Coef** | **95% CI** | **Coef** | **95% CI** | **Coef** | **95% CI** |
| First marriage (ref category) |  |  |  |  |  |  |  |  |
| Remarriage | 0.55 | (-0.41, 1.52) | -0.03 | (-1.06, 1.01) | **-0.92** | **(-1.71, -0.12)** | 0.09 | (-0.39, 0.57) |
| Divorced / separated | -1.17 | (-3.24, 0.90) | 0.02 | (-0.89, 0.94) | **0.95** | **(0.23, 1.67)** | 0.34 | (-0.12, 0.80) |
| Widowed | 0.57 | (-0.43, 1.58) | 0.71 | (-0.17, 1.58) | -0.23 | (-1.25, 0.79) | **0.45** | **(0.07, 0.83)** |
| Never married | 0.42 | (-1.08, 1.91) | 0.29 | (-0.96, 1.55) | -0.99 | (-2.51, 0.53) | 0.14 | (-0.74, 1.03) |

*p<0.05 in bold*

S1D Table: Logistic regression coefficients showing the likelihood of unable to do the walking speed test due to health reasons by marital status adjusted for age

|  | ELSA | | | | HRS | | | |
| --- | --- | --- | --- | --- | --- | --- | --- | --- |
|  | **Men** | | **Women** | | **Men** | | **Women** | |
|  | **Coef** | **95% CI** | **Coef** | **95% CI** | **Coef** | **95% CI** | **Coef** | **95% CI** |
| First marriage (ref category) |  |  |  |  |  |  |  |  |
| Remarriage | 0.58 | (-0.10, 1.27) | 0.19 | (-0.65, 1.03) | 0.37 | (-0.09, 0.83) | -0.08 | (-0.62, 0.45) |
| Divorced / separated | 0.48 | (-0.46, 1.41) | **0.64** | **(0.06, 1.22)** | **0.86** | **(0.30, 1.42)** | **0.97** | **(0.55, 1.40)** |
| Widowed | 0.20 | (-0.49, 0.88) | 0.24 | (-0.22, 0.71) | **0.69** | **(0.23, 1.14)** | **0.73** | **(0.40, 1.07)** |
| Never married | 0.34 | (-0.74, 1.42) | 0.43 | (-0.34, 1.20) | -0.42 | (-1.95, 1.11) | 0.41 | (-0.37, 1.20) |

*p<0.05 in bold*

### Sensitivity analysis of assigning different values to those who were unable to do the test due to health reasons

S1E Table: Grip strength, men in ELSA

|  | Age specific lowest quintile given to unables | | Complete cases - excluding unables | | Age specific mean score given to unables | | Age specific lowest score given to unables | |
| --- | --- | --- | --- | --- | --- | --- | --- | --- |
|  | **Coef** | **95% CI** | **Coef** | **95% CI** | **Coef** | **95% CI** | **Coef** | **95% CI** |
| Marital status (first marriage ref category) |  |  |  |  |  |  |  |  |
| Remarried | **0.72** | **(0.27, 1.16)** | **0.77** | **(0.32, 1.22)** | **0.64** | **(0.15, 1.12)** | **0.63** | **(0.15, 1.12)** |
| Divorced / separated | 0.52 | (-0.02, 1.07) | 0.49 | (-0.05, 1.03) | **0.63** | **(0.04, 1.22)** | **0.63** | **(0.04, 1.22)** |
| Widowed | -0.40 | (-1.01, 0.21) | -0.37 | (-0.98, 0.24) | -0.54 | (-1.20, 0.12) | -0.54 | (-1.21, 0.12) |
| Never married | -0.13 | (-0.88, 0.61) | -0.12 | (-0.87, 0.63) | -0.17 | (-0.99, 0.64) | -0.17 | (-0.99, 0.64) |

*p<0.05 in bold*

*Adjusted for age, ethnicity, work status, parental status, education, wealth, smoking status, physical activity and BMI, self-rated health, chronic health conditions and psychological morbidity*

S1F Table: Grip strength, women in ELSA

|  | Age specific lowest quintile given to unables | | Complete cases - excluding unables | | Mean score given to unables | | Lowest score given to unables | |
| --- | --- | --- | --- | --- | --- | --- | --- | --- |
|  | **Coef** | **95% CI** | **Coef** | **95% CI** | **Coef** | **95% CI** | **Coef** | **95% CI** |
| Marital status (first marriage ref category) |  |  |  |  |  |  |  |  |
| Remarried | **0.36** | **(0.03, 0.69)** | **0.37** | **(0.04, 0.70)** | **0.36** | **(0.00, 0.73)** | **0.36** | **(0.00, 0.73)** |
| Divorced / separated | 0.04 | (-0.28, 0.37) | 0.06 | (-0.26, 0.39) | 0.02 | (-0.33, 0.38) | 0.02 | (-0.34, 0.38) |
| Widowed | 0.01 | (-0.29, 0.32) | 0.06 | (-0.25, 0.36) | -0.09 | (-0.42, 0.24) | -0.09 | (-0.42, 0.24) |
| Never married | -0.27 | (-0.79, 0.26) | -0.24 | (-0.77, 0.29) | -0.26 | (-0.84, 0.32) | -0.26 | (-0.84, 0.33) |

*p<0.05 in bold*

*Adjusted for age, ethnicity, work status, parental status, education, wealth, smoking status, physical activity and BMI, self-rated health, chronic health conditions and psychological morbidity*

S1G Table: Grip strength men in HRS

|  | Age specific lowest quintile given to unables | | Complete cases - excluding unables | | Mean score given to unables | | Lowest score given to unables | |
| --- | --- | --- | --- | --- | --- | --- | --- | --- |
|  | **Coef** | **95% CI** | **Coef** | **95% CI** | **Coef** | **95% CI** | **Coef** | **95% CI** |
| Marital status (first marriage ref category) |  |  |  |  |  |  |  |  |
| Remarried | **0.31** | **(0.03, 0.59)** | **0.30** | **(0.02, 0.58)** | **0.30** | **(0.02, 0.58)** | **0.34** | **(0.02, 0.58)** |
| Divorced / separated | -0.05 | (-0.45, 0.35) | 0.05 | (-0.36, 0.45) | 0.06 | (-0.33, 0.46) | -0.31 | (-0.33, 0.46) |
| Widowed | -0.41 | (-0.85, 0.03) | -0.44 | (-0.88, 0.01) | -0.44 | (-0.87, 0.00) | -0.37 | (-0.87, 0.00) |
| Never married | **-0.97** | **(-1.77, -0.18)** | **-0.96** | **(-1.76, -0.16)** | **-0.96** | **(-1.75, -0.17)** | **-0.99** | **(-1.75, -0.17)** |

*p<0.05 in bold*

*Adjusted for age, ethnicity, work status, parental status, education, wealth, smoking status, physical activity and BMI, self-rated health, chronic health conditions and psychological morbidity*

S1H Table: Grip strength, women in HRS

|  | Age specific lowest quintile given to unables | | Complete cases - excluding unables | | Mean score given to unables | | Lowest score given to unables | |
| --- | --- | --- | --- | --- | --- | --- | --- | --- |
|  | **Coef** | **95% CI** | **Coef** | **95% CI** | **Coef** | **95% CI** | **Coef** | **95% CI** |
| Marital status (first marriage ref category) |  |  |  |  |  |  |  |  |
| Remarried | -0.01 | (-0.24, 0.21) | 0.02 | (-0.20, 0.25) | 0.00 | (-0.21, 0.22) | -0.05 | (-0.32, 0.23) |
| Divorced / separated | 0.04 | (-0.21, 0.29) | 0.06 | (-0.19, 0.31) | 0.06 | (-0.18, 0.30) | -0.01 | (-0.31, 0.29) |
| Widowed | -0.20 | (-0.41, 0.01) | -0.17 | (-0.37, 0.04) | -0.16 | (-0.36, 0.04) | **-0.30** | **(-0.55, -0.04)** |
| Never married | 0.24 | (-0.26, 0.74) | 0.24 | (-0.27, 0.74) | 0.21 | (-0.27, 0.70) | 0.26 | (-0.36, 0.88) |

*p<0.05 in bold*

*Adjusted for age, ethnicity, work status, parental status, education, wealth, smoking status, physical activity and BMI, self-rated health, chronic health conditions and psychological morbidity*

S1I Table: Walking speed, men in ELSA

|  | Age specific lowest quintile given to unables | | Complete cases - excluding unables | | Mean score given to unables | | Lowest score given to unables | |
| --- | --- | --- | --- | --- | --- | --- | --- | --- |
|  | **Coef** | **95% CI** | **Coef** | **95% CI** | **Coef** | **95% CI** | **Coef** | **95% CI** |
| Marital status (first marriage ref category) |  |  |  |  |  |  |  |  |
| Remarried | 0.014 | (-0.020, 0.048) | 0.022 | (-0.013, 0.057) | 0.022 | (-0.011, 0.056) | 0.008 | (-0.029, 0.045) |
| Divorced / separated | -0.015 | (-0.061, 0.032) | -0.014 | (-0.062, 0.033) | -0.009 | (-0.055, 0.037) | -0.020 | (-0.070, 0.030) |
| Widowed | **-0.042** | **(-0.077, -0.006)** | **-0.046** | **(-0.082, -0.010)** | **-0.041** | **(-0.076, -0.006)** | **-0.042** | **(-0.08, -0.004)** |
| Never married | **-0.082** | **(-0.147, -0.017)** | **-0.081** | **(-0.147, -0.015)** | **-0.075** | **(-0.139, -0.010)** | **-0.088** | **(-0.159, -0.018)** |

*p<0.05 in bold*

*Adjusted for age, ethnicity, work status, parental status, education, wealth, smoking status, physical activity and BMI, self-rated health, chronic health conditions and psychological morbidity*

S1J Table: Walking speed, women in ELSA

|  | Age specific lowest quintile given to unables | | Complete cases - excluding unables | | Mean score given to unables | | Lowest score given to unables | |
| --- | --- | --- | --- | --- | --- | --- | --- | --- |
|  | **Coef** | **95% CI** | **Coef** | **95% CI** | **Coef** | **95% CI** | **Coef** | **95% CI** |
| Marital status (first marriage ref category) |  |  |  |  |  |  |  |  |
| Remarried | 0.020 | (-0.017, 0.056) | 0.004 | (-0.019, 0.056) | 0.017 | (-0.019, 0.053) | 0.022 | (-0.020, 0.060) |
| Divorced / separated | -0.002 | (-0.036, 0.032) | 0.063 | (-0.029, 0.041) | 0.007 | (-0.026, 0.041) | -0.011 | (-0.050, 0.030) |
| Widowed | 0.000 | (-0.024, 0.024) | -0.156 | (-0.025, 0.024) | 0.002 | (-0.022, 0.025) | -0.002 | (-0.030, 0.030) |
| Never married | -0.033 | (-0.087, 0.022) | 0.214 | (-0.088, 0.026) | -0.027 | (-0.081, 0.026) | -0.036 | (-0.100, 0.030) |

*p<0.05 in bold*

*Adjusted for age, ethnicity, work status, parental status, education, wealth, smoking status, physical activity and BMI, self-rated health, chronic health conditions and psychological morbidity*

S1K Table: Walking speed men in HRS

|  | Age specific lowest quintile given to unables | | Complete cases - excluding unables | | Mean score given to unables | | Lowest score given to unables | |
| --- | --- | --- | --- | --- | --- | --- | --- | --- |
|  | **Coef** | **95% CI** | **Coef** | **95% CI** | **Coef** | **95% CI** | **Coef** | **95% CI** |
| Marital status (first marriage ref category) |  |  |  |  |  |  |  |  |
| Remarried | 0.016 | (-0.003, 0.035) | **0.019** | **(0.000, 0.039)** | **0.020** | **(0.002, 0.039)** | 0.011 | (-0.010, 0.032) |
| Divorced / separated | -0.002 | (-0.031, 0.027) | 0.003 | (-0.027, 0.033) | 0.004 | (-0.025, 0.033) | -0.010 | (-0.043, 0.022) |
| Widowed | **-0.037** | **(-0.062, -0.011)** | **-0.033** | **(-0.060, -0.007)** | **-0.027** | **(-0.052, -0.002)** | **-0.046** | **(-0.074, -0.018)** |
| Never married | -0.021 | (-0.083, 0.041) | -0.026 | (-0.090, 0.037) | -0.040 | (-0.101, 0.021) | -0.002 | (-0.071, 0.067) |

*p<0.05 in bold*

*Adjusted for age, ethnicity, work status, parental status, education, wealth, smoking status, physical activity and BMI, self-rated health, chronic health conditions and psychological morbidity*

S1L Table: Walking speed women in HRS

|  | Age specific lowest quintile given to unables | | Complete cases - excluding unables | | Mean score given to unables | | Lowest score given to unables | |
| --- | --- | --- | --- | --- | --- | --- | --- | --- |
|  | **Coef** | **95% CI** | **Coef** | **95% CI** | **Coef** | **95% CI** | **Coef** | **95% CI** |
| Marital status (first marriage ref category) |  |  |  |  |  |  |  |  |
| Remarried | 0.015 | (-0.006, 0.036) | 0.013 | (-0.008, 0.034) | 0.012 | (-0.008, 0.033) | 0.017 | (-0.006, 0.040) |
| Divorced / separated | 0.001 | (-0.022, 0.023) | 0.009 | (-0.015, 0.032) | 0.008 | (-0.014, 0.030) | -0.006 | (-0.032, 0.019) |
| Widowed | -0.008 | (-0.024, 0.007) | -0.005 | (-0.021, 0.011) | -0.006 | (-0.021, 0.010) | -0.010 | (-0.028, 0.007) |
| Never married | -0.028 | (-0.074, 0.019) | -0.023 | (-0.072, 0.026) | -0.015 | (-0.061, 0.031) | -0.040 | (-0.092, 0.012) |

*p<0.05 in bold*

*Adjusted for age, ethnicity, work status, parental status, education, wealth, smoking status, physical activity and BMI, self-rated health, chronic health conditions and psychological morbidity*
