# Supplementary material for: Marriage and physical capability at mid to later life in England and the USA
Source: PLoS One. 2019 Jan 23;14(1):e0209388. doi: 10.1371/journal.pone.0209388 (PMC6343866; doi:10.1371/journal.pone.0209388)
Supplement: S2 File — (DOCX) [file pone.0209388.s002.docx]

## S2: comparison of the complete sample with the analytic sample

S2A and S2B Tables show the mean age adjusted grip strength and walking speed for the analytic sample and the sample containing the cases with missing data for ELSA and HRS.

The final analytic sample and the complete sample had comparable mean grip strength for men and women on both ELSA and HRS. There were also few significant differences in the age adjusted mean grip strength by marital status between the final analytic sample and the complete sample, although in ELSA widowed women in the final analytic sample had a stronger grip strength than widowed women in the complete sample. There were no differences by marital status between the analytic sample and the complete sample for the measure of grip strength in HRS.

S2A Table: Comparison of age adjusted grip strength estimates by marital status between the analytical sample and the complete sample in ELSA and HRS

|  | ELSA | | | | | | | |
| --- | --- | --- | --- | --- | --- | --- | --- | --- |
|  | **Men** | | | | **Women** | | | |
|  | **Complete sample** | | **Final analytic sample** | | **Complete sample** | | **Final analytic sample** | |
|  | **Mean**  **(kg/m)** | **95% CI** | **Mean**  **(kg/m)** | **95% CI** | **Mean**  **(kg/m)** | **95% CI** | **Mean**  **(kg/m)** | **95% CI** |
| Overall Mean | 22.90 | (22.76, 23.03) | 22.89 | (22.75, 23.04) | 14.82 | (14.69, 14.94) | 14.83 | (14.70, 14.95) |
| First marriage | 23.05 | (22.85, 23.25) | 23.07 | (22.86, 23.27) | 14.91 | (14.76, 15.06) | 14.89 | (14.74, 15.05) |
| Remarried | 23.67 | (23.27, 24.08) | 23.68 | (23.26, 24.10) | 15.07 | (14.77, 15.37) | 15.07 | (14.76, 15.38) |
| Divorced / separated | 22.86 | (22.37, 23.35) | 22.80 | (22.29, 23.30) | 14.54 | (14.27, 14.81) | 14.50 | (14.22, 14.78) |
| Widowed | 22.14 | (21.56, 22.73) | 22.17 | (21.58, 22.77) | 14.51 | (14.26, 14.75) | **14.56** | **(14.31, 14.82)** |
| Never married | 21.54 | (20.93, 22.15) | 21.60 | (20.98, 22.23) | 14.46 | (14.00, 14.91) | 14.53 | (14.07, 15.00) |
| *Total (N)* | *3,559* | | *3,382* | | *4,302* | | *4,096* | |
|  | **HRS** | | | | | | | |
|  | **Men** | | | | **Women** | | | |
|  | **Complete sample** | | **Final analytic sample** | | **Complete sample** | | **Final analytic sample** | |
|  | **Mean**  **(kg/m)** | **95% CI** | **Mean**  **(kg/m)** | **95% CI** | **Mean**  **(kg/m)** | **95% CI** | **Mean**  **(kg/m)** | **95% CI** |
| Overall Mean | 22.82 | (22.71, 22.92) | 22.83 | (22.72, 22.94) | 15.07 | (14.98, 15.16) | 15.08 | (14.99, 15.17) |
| First marriage | 23.01 | (22.85, 23.18) | 23.02 | (22.85, 23.19) | 15.19 | (15.06, 15.32) | 15.20 | (15.07, 15.33) |
| Remarried | 23.26 | (23.02, 23.51) | 23.28 | (23.04, 23.52) | 15.14 | (14.94, 15.34) | 15.15 | (14.95, 15.35) |
| Divorced / separated | 22.24 | (21.88, 22.61) | 22.23 | (21.86, 22.6) | 15.06 | (14.86, 15.27) | 15.10 | (14.90, 15.31) |
| Widowed | 22.07 | (21.65, 22.5) | 22.08 | (21.66, 22.51) | 14.81 | (14.65, 14.96) | 14.81 | (14.65, 14.97) |
| Never married | 20.68 | (19.99, 21.37) | 20.74 | (20.04, 21.44) | 15.31 | (14.86, 15.75) | 15.29 | (14.84, 15.74) |
| *Total (N)* | *5,502* | | *5,406* | | *7,530* | | *7,345* | |

*p<0.05 shown in bold*

*Tests of significance were carried out by running a linear regression on grip strength and marital status adjusted for age, with interactions between marital status and variable which flagged whether cases was in the analytic sample or not.*

For both men and women in ELSA the final analytic sample had an overall faster mean walking speed than the complete sample (*p*<0.001), but there were no differences in walking speed by marital status between the two samples. Among men in HRS the two samples had comparable walking speeds, but among women the analytic sample had an overall faster walking speed. There was one difference between the two samples by marital status. In HRS widowed men in the final analytic sample had a slower walking speed than the sample containing the missing data, 0.717 m/s in the analytic sample compared to 0.723 m/s in the complete sample.

Overall there were few marital status differences between the complete sample and the analytic sample. Where there were differences between the two samples could result in underestimation of grip strength differences between widowed women and women in their first marriage in ELSA, whilst for the measure of walking speed any differences between widowed men and men in their first marriage could be overestimated in HRS.

S2B Table: Comparison of age adjusted walking speed estimates by marital status between analytical sample and the complete sample in ELSA and HRS

|  | ELSA | | | | | | | |
| --- | --- | --- | --- | --- | --- | --- | --- | --- |
|  | **Men** | | | | **Women** | | | |
|  | **Complete sample** | | **Final analytic sample** | | **Complete sample** | | **Final analytic sample** | |
|  | **Mean**  **(m/s)** | **95% CI** | **Mean**  **(m/s)** | **95% CI** | **Mean**  **(m/s)** | **95% CI** | **Mean**  **(m/s)** | **95% CI** |
| Overall mean | 0.826 | (0.815, 0.838) | **0.857** | (0.844, 0.870) | 0.752 | (0.742, 0.763) | **0.782** | **(0.770, 0.793)** |
| First marriage | 0.861 | (0.845, 0.876) | 0.883 | (0.867, 0.900) | 0.770 | (0.754, 0.787) | 0.803 | (0.785, 0.821) |
| Remarried | 0.847 | (0.816, 0.879) | 0.879 | (0.844, 0.914) | 0.748 | (0.712, 0.784) | 0.778 | (0.738, 0.818) |
| Divorced / separated | 0.753 | (0.710, 0.796) | 0.795 | (0.745, 0.844) | 0.731 | (0.700, 0.762) | 0.753 | (0.719, 0.788) |
| Widowed | 0.792 | (0.760, 0.823) | 0.817 | (0.781, 0.853) | 0.727 | (0.710, 0.744) | 0.759 | (0.739, 0.779) |
| Never married | 0.741 | (0.691, 0.791) | 0.776 | (0.716, 0.835) | 0.719 | (0.673, 0.765) | 0.752 | (0.699, 0.805) |
| *Total (N)* | *1,808* | | *1,630* | | *2,236* | | *2,015* | |
|  | **HRS** | | | | | | | |
|  | **Men** | | | | **Women** | | | |
|  | **Complete sample** | | **Final analytic sample** | | **Complete sample** | | **Final analytic sample** | |
|  | **Mean**  **(m/s)** | **95% CI** | **Mean**  **(m/s)** | **95% CI** | **Mean**  **(m/s)** | **95% CI** | **Mean**  **(m/s)** | **95% CI** |
| Overall mean | 0.780 | (0.771, 0.788) | 0.780 | (0.771, 0.788) | 0.697 | (0.690, 0.704) | **0.699** | **(0.691, 0.706)** |
| First marriage | 0.798 | (0.787, 0.810) | 0.799 | (0.787, 0.811) | 0.730 | (0.718, 0.742) | 0.731 | (0.719, 0.743) |
| Remarried | 0.799 | (0.782, 0.816) | 0.800 | (0.783, 0.818) | 0.736 | (0.716, 0.756) | 0.736 | (0.716, 0.756) |
| Divorced / separated | 0.737 | (0.708, 0.765) | 0.737 | (0.708, 0.766) | 0.662 | (0.641, 0.682) | 0.666 | (0.644, 0.687) |
| Widowed | 0.723 | (0.699, 0.748) | **0.717** | **(0.692, 0.742)** | 0.664 | (0.653, 0.676) | 0.666 | (0.654, 0.677) |
| Never married | 0.718 | (0.660, 0.775) | 0.718 | (0.660, 0.776) | 0.657 | (0.611, 0.702) | 0.657 | (0.611, 0.703) |
| *Total (N)* | *3,608* | | *3,541* | | *4,915* | | *4,796* | |

*p<0.05 shown in bold*

*Tests of significance were carried out by running a linear regression on walking speed and marital status adjusted for age, with interactions between marital status and a variable which flagged whether cases was in the analytic sample or not.*
