# Supplementary material for: Marriage and physical capability at mid to later life in England and the USA
Source: PLoS One. 2019 Jan 23;14(1):e0209388. doi: 10.1371/journal.pone.0209388 (PMC6343866; doi:10.1371/journal.pone.0209388)
Supplement: S3 File — (DOCX) [file pone.0209388.s003.docx]

## S3: Models containing all covariates

### Grip Strength

S3A Table: Grip strength models with coefficients for all covariates, men in ELSA

|  | Model1 | | Model 2 | | Model 3 | |
| --- | --- | --- | --- | --- | --- | --- |
|  | **Coef** | **95% CI** | **Coef** | **95% CI** | **Coef** | **95% CI** |
| Marital status (first marriage ref category) |  |  |  |  |  |  |
| Remarried | **0.61** | **(0.15, 1.07)** | **0.76** | **(0.30, 1.22)** | **0.72** | **(0.27, 1.16)** |
| Divorced / separated | -0.10 | (-0.63, 0.44) | 0.48 | (-0.07, 1.03) | 0.52 | (-0.02, 1.07) |
| Widowed | **-0.73** | **(-1.36, -0.11)** | -0.43 | (-1.05, 0.19) | -0.40 | (-1.01, 0.21) |
| Never married | -0.61 | (-1.38, 0.15) | -0.26 | (-1.02, 0.51) | -0.13 | (-0.88, 0.61) |
| Age (50-59 ref category) |  |  |  |  |  |  |
| 60-69 | **-1.48** | **(-1.9, -1.06)** | **-1.60** | **(-2.02, -1.18)** | **-1.59** | **(-2, -1.17)** |
| 70-79 | **-3.66** | **(-4.19, -3.14)** | **-3.80** | **(-4.32, -3.27)** | **-3.65** | **(-4.18, -3.12)** |
| 80+ | **-6.98** | **(-7.7, -6.27)** | **-7.07** | **(-7.78, -6.35)** | **-6.46** | **(-7.19, -5.73)** |
| Ethnicity (white ref category) |  |  |  |  |  |  |
| Non-white | **-2.30** | **(-3.24, -1.36)** | **-2.13** | **(-3.07, -1.19)** | **-1.51** | **(-2.43, -0.58)** |
| Work status (working ref category) |  |  |  |  |  |  |
| Not working | **-1.72** | **(-2.11, -1.33)** | **-1.58** | **(-1.97, -1.19)** | **-1.09** | **(-1.48, -0.69)** |
| Parental status (has children ref category) |  |  |  |  |  |  |
| No children | **-0.87** | **(-1.38, -0.36)** | **-0.88** | **(-1.39, -0.38)** | **-0.75** | **(-1.25, -0.26)** |
| Education (low ref category) |  |  |  |  |  |  |
| Medium |  |  | 0.09 | (-0.28, 0.46) | -0.02 | (-0.38, 0.35) |
| High |  |  | -0.28 | (-0.75, 0.20) | -0.32 | (-0.79, 0.15) |
| Wealth (lowest wealth quintile ref category) |  |  |  |  |  |  |
| 2^nd^ |  |  | **0.64** | **(0.08, 1.20)** | 0.35 | (-0.21, 0.90) |
| 3^rd^ |  |  | **1.47** | **(0.91, 2.03)** | **0.90** | **(0.34, 1.46)** |
| 4^th^ |  |  | **1.77** | **(1.22, 2.33)** | **1.20** | **(0.63, 1.76)** |
| 5^th^ high wealth |  |  | **1.94** | **(1.36, 2.51)** | **1.20** | **(0.61, 1.78)** |
| Smoking status (never smoked ref category) |  |  |  |  |  |  |
| Former smoker |  |  |  |  | 0.27 | (-0.07, 0.62) |
| Current smoker |  |  |  |  | 0.15 | (-0.38, 0.69) |
| Physical activity (moderate activity ref category) |  |  |  |  |  |  |
| Sedentary |  |  |  |  | **-1.64** | **(-2.41, -0.86)** |
| Low |  |  |  |  | **-0.62** | **(-1.06, -0.17)** |
| High |  |  |  |  | **0.65** | **(0.27, 1.03)** |
| Body Mass Index (BMI<25 ref category) |  |  |  |  |  |  |
| Overweight BMI (25 -29) |  |  |  |  | **1.25** | **(0.85, 1.64)** |
| Obese BMI (30+) |  |  |  |  | **1.86** | **(1.41, 2.32)** |
| Self-rated health (excellent / v. good ref category) |  |  |  |  |  |  |
| Good |  |  |  |  | -0.30 | (-0.67, 0.06) |
| Fair / poor |  |  |  |  | **-1.22** | **(-1.69, -0.75)** |
| Chronic health conditions (0 reported conditions ref category) |  |  |  |  |  |  |
| Reported 1 condition |  |  |  |  | -0.08 | (-0.47, 0.30) |
| Reported 2 conditions |  |  |  |  | **-0.53** | **(-0.99, -0.08)** |
| Reported 3+ conditions |  |  |  |  | **-0.73** | **(-1.28, -0.18)** |
| CES-D (CES-D<3 ref category) |  |  |  |  |  |  |
| CES-D≥3 |  |  |  |  | -0.22 | (-0.69, 0.26) |
| *Constant* | *26.25* | *(25.91, 26.59)* | *24.85* | *(24.27, 25.42)* | *24.32* | *(23.56, 25.07)* |

*Results p<0.05 are shown in bold.*

*Model 1: Age and demographics (ethnicity, work status and parental status)*

*Model 2: Age + demographic and socio-economic measures (education and wealth)*

*Model 3: Age + demographic and socio-economic measures + health behaviours (smoking status, physical activity and BMI) + physical health and psychological morbidity (self-rated health, chronic health conditions and psychological morbidity)*

S3B Table: Grip strength models with coefficients for all covariates, men in HRS

|  | Model1 | | Model 2 | | Model 3 | |
| --- | --- | --- | --- | --- | --- | --- |
|  | **Coef** | **95% CI** | **Coef** | **95% CI** | **Coef** | **95% CI** |
| Marital status (first marriage ref category) |  |  |  |  |  |  |
| Remarried | 0.22 | (-0.08, 0.51) | **0.30** | **(0.01, 0.59)** | **0.31** | **(0.03, 0.59)** |
| Divorced / separated | **-0.62** | **(-1.02, -0.21)** | -0.20 | (-0.61, 0.21) | -0.05 | (-0.45, 0.35) |
| Widowed | **-0.80** | **(-1.26, -0.35)** | **-0.53** | **(-0.99, -0.07)** | -0.41 | (-0.85, 0.03) |
| Never married | **-1.45** | **(-2.28, -0.62)** | **-1.07** | **(-1.89, -0.24)** | **-0.97** | **(-1.77, -0.18)** |
| Age (50-59 ref category) |  |  |  |  |  |  |
| 60-69 | **-2.16** | **(-2.51, -1.80)** | **-2.30** | **(-2.65, -1.94)** | **-2.07** | **(-2.41, -1.72)** |
| 70-79 | **-4.33** | **(-4.71, -3.94)** | **-4.50** | **(-4.89, -4.11)** | **-4.00** | **(-4.4, -3.61)** |
| 80+ | **-7.68** | **(-8.15, -7.20)** | **-7.92** | **(-8.4, -7.44)** | **-6.82** | **(-7.3, -6.33)** |
| Ethnicity (white ref category) |  |  |  |  |  |  |
| Hispanic | **-1.64** | **(-2.08, -1.2)** | **-1.22** | **(-1.66, -0.77)** | **-1.22** | **(-1.66, -0.79)** |
| Black | **-0.55** | **(-0.93, -0.16)** | -0.09 | (-0.49, 0.30) | -0.01 | (-0.39, 0.37) |
| Other | **-1.26** | **(-2.29, -0.24)** | -0.99 | (-2.00, 0.03) | **-1.04** | **(-2.01, -0.06)** |
| Work status (working ref category) |  |  |  |  |  |  |
| Not working | **-1.42** | **(-1.71, -1.12)** | **-1.33** | **(-1.62, -1.04)** | **-0.72** | **(-1.01, -0.43)** |
| Parental status (has children ref category) |  |  |  |  |  |  |
| No children | **-1.03** | **(-1.63, -0.43)** | **-1.10** | **(-1.70, -0.51)** | **-1.03** | **(-1.60, -0.46)** |
| Education (low ref category) |  |  |  |  |  |  |
| Medium |  |  | **0.34** | **(0.02, 0.67)** | 0.22 | (-0.09, 0.53) |
| High |  |  | -0.22 | (-0.52, 0.09) | **-0.40** | **(-0.70, -0.10)** |
| Wealth (lowest wealth quintile ref category) |  |  |  |  |  |  |
| 2nd |  |  | **0.75** | **(0.33, 1.18)** | **0.45** | **(0.05, 0.86)** |
| 3rd |  |  | **1.44** | **(1.02, 1.85)** | **0.91** | **(0.5, 1.31)** |
| 4th |  |  | **1.73** | **(1.3, 2.16)** | **1.10** | **(0.68, 1.52)** |
| 5th high wealth |  |  | **1.92** | **(1.49, 2.36)** | **1.26** | **(0.83, 1.69)** |
| Smoking status (never smoked ref category) |  |  |  |  |  |  |
| Former smoker |  |  |  |  | 0.09 | (-0.17, 0.35) |
| Current smoker |  |  |  |  | 0.21 | (-0.16, 0.59) |
| Physical activity (moderate activity ref category) |  |  |  |  |  |  |
| Sedentary |  |  |  |  | **-2.81** | **(-3.34, -2.28)** |
| Low |  |  |  |  | **-0.79** | **(-1.10, -0.47)** |
| High |  |  |  |  | 0.16 | (-0.11, 0.43) |
| Body Mass Index (BMI<25 ref category) |  |  |  |  |  |  |
| Overweight BMI (25 -29) |  |  |  |  | **1.18** | **(0.89, 1.47)** |
| Obese BMI (30+) |  |  |  |  | **1.99** | **(1.68, 2.31)** |
| Self-rated health (excellent / v. good ref category) |  |  |  |  |  |  |
| Good |  |  |  |  | -0.27 | (-0.55, 0.02) |
| Fair / poor |  |  |  |  | **-0.90** | **(-1.24, -0.57)** |
| Chronic health conditions (0 reported conditions ref category) |  |  |  |  |  |  |
| Reported 1 condition |  |  |  |  | -0.38 | (-0.76, 0.01) |
| Reported 2 conditions |  |  |  |  | **-1.06** | **(-1.46, -0.66)** |
| Reported 3+ conditions |  |  |  |  | **-1.58** | **(-1.99, -1.17)** |
| CES-D (CES-D<3 ref category) |  |  |  |  |  |  |
| CES-D≥3 |  |  |  |  | **-0.46** | **(-0.79, -0.12)** |
| *Constant* | *27.39* | *(27.08, 27.7)* | *26.00* | *(25.54, 26.47)* | *26.09* | *(25.48, 26.71)* |

*Results p<0.05 are shown in bold.*

*Model 1: Age and demographics (ethnicity, work status and parental status)*

*Model 2: Age + demographic and socio-economic measures (education and wealth)*

*Model 3: Age + demographic and socio-economic measures + health behaviours (smoking status, physical activity and BMI) + physical health and psychological morbidity (self-rated health, chronic health conditions and psychological morbidity)*

S3C Table: Grip strength models with coefficients for all covariates, women in ELSA

|  | Model1 | | Model 2 | | Model 3 | |
| --- | --- | --- | --- | --- | --- | --- |
|  | **Coef** | **95% CI** | **Coef** | **95% CI** | **Coef** | **95% CI** |
| Marital status (first marriage ref category) |  |  |  |  |  |  |
| Remarried | 0.14 | (-0.21, 0.48) | 0.29 | (-0.05, 0.63) | 0.36 | (0.03, 0.69) |
| Divorced / separated | **-0.42** | **(-0.74, -0.10)** | -0.06 | (-0.39, 0.27) | 0.04 | (-0.28, 0.37) |
| Widowed | -0.31 | (-0.61, 0.00) | -0.08 | (-0.39, 0.23) | 0.01 | (-0.29, 0.32) |
| Never married | -0.48 | (-1.02, 0.07) | -0.31 | (-0.86, 0.24) | -0.27 | (-0.79, 0.26) |
| Age (50-59 ref category) |  |  |  |  |  |  |
| 60-69 | **-0.77** | **(-1.06, -0.48)** | **-0.78** | **(-1.07, -0.49)** | **-0.78** | **(-1.07, -0.50)** |
| 70-79 | **-2.44** | **(-2.79, -2.08)** | **-2.36** | **(-2.71, -2.01)** | **-2.06** | **(-2.41, -1.71)** |
| 80+ | **-4.91** | **(-5.38, -4.44)** | **-4.79** | **(-5.26, -4.33)** | **-3.97** | **(-4.45, -3.49)** |
| Ethnicity (white ref category) |  |  |  |  |  |  |
| Non-white | -0.45 | (-1.14, 0.24) | -0.47 | (-1.16, 0.21) | -0.10 | (-0.77, 0.57) |
| Work status (working ref category) |  |  |  |  |  |  |
| Not working | **-0.91** | **(-1.19, -0.63)** | **-0.82** | **(-1.1, -0.54)** | **-0.43** | **(-0.71, -0.16)** |
| Parental status (has children ref category) |  |  |  |  |  |  |
| No children | 0.20 | (-0.13, 0.52) | 0.08 | (-0.24, 0.41) | 0.13 | (-0.19, 0.44) |
| Education (low ref category) |  |  |  |  |  |  |
| Medium |  |  | **0.25** | **(0.01, 0.49)** | 0.15 | (-0.08, 0.39) |
| High |  |  | **0.65** | **(0.30, 0.99)** | **0.46** | **(0.12, 0.79)** |
| Wealth (lowest wealth quintile ref category) |  |  |  |  |  |  |
| 2^nd^ |  |  | 0.10 | (-0.25, 0.46) | -0.05 | (-0.39, 0.29) |
| 3^rd^ |  |  | **0.51** | **(0.15, 0.86)** | 0.20 | (-0.15, 0.55) |
| 4^th^ |  |  | **0.76** | **(0.39, 1.12)** | **0.38** | **(0.02, 0.74)** |
| 5^th^ high wealth |  |  | **1.02** | **(0.64, 1.40)** | **0.58** | **(0.20, 0.96)** |
| Smoking status (never smoked ref category) |  |  |  |  |  |  |
| Former smoker |  |  |  |  | 0.18 | (-0.04, 0.41) |
| Current smoker |  |  |  |  | **0.82** | **(0.49, 1.16)** |
| Physical activity (moderate activity ref category) |  |  |  |  |  |  |
| Sedentary |  |  |  |  | **-1.49** | **(-1.99, -1.00)** |
| Low |  |  |  |  | **-0.63** | **(-0.88, -0.37)** |
| High |  |  |  |  | **0.58** | **(0.29, 0.87)** |
| Body Mass Index (BMI<25 ref category) |  |  |  |  |  |  |
| Overweight BMI (25 -29) |  |  |  |  | **0.46** | **(0.20, 0.71)** |
| Obese BMI (30+) |  |  |  |  | **0.95** | **(0.68, 1.21)** |
| Self-rated health (excellent / v. good ref category) |  |  |  |  |  |  |
| Good |  |  |  |  | **-0.53** | **(-0.78, -0.28)** |
| Fair / poor |  |  |  |  | **-1.08** | **(-1.40, -0.76)** |
| Chronic health conditions (0 reported conditions ref category) |  |  |  |  |  |  |
| Reported 1 condition |  |  |  |  | -0.11 | (-0.38, 0.16) |
| Reported 2 conditions |  |  |  |  | **-0.45** | **(-0.76, -0.14)** |
| Reported 3+ conditions |  |  |  |  | **-0.74** | **(-1.11, -0.38)** |
| CES-D (CES-D<3 ref category) |  |  |  |  |  |  |
| CES-D≥3 |  |  |  |  | **-0.54** | **(-0.80, -0.29)** |
| *Constant* | *16.86* | *(16.62, 17.1)* | *15.96* | *(15.58, 16.34)* | *16.14* | *(15.67, 16.61)* |

*Results p<0.05 are shown in bold.*

*Model 1: Age and demographics (ethnicity, work status and parental status)*

*Model 2: Age + demographic and socio-economic measures (education and wealth)*

*Model 3: Age + demographic and socio-economic measures + health behaviours (smoking status, physical activity and BMI) + physical health and psychological morbidity (self-rated health, chronic health conditions and psychological morbidity)*

S3D Table: Grip strength models with coefficients for all covariates, women in HRS

|  | Model1 | | Model 2 | | Model 3 | |
| --- | --- | --- | --- | --- | --- | --- |
|  | **Coef** | **95% CI** | **Coef** | **95% CI** | **Coef** | **95% CI** |
| Marital status (first marriage ref category) |  |  |  |  |  |  |
| Remarried | -0.11 | (-0.34, 0.12) | -0.04 | (-0.27, 0.19) | -0.01 | (-0.24, 0.21) |
| Divorced / separated | **-0.29** | **(-0.54, -0.05)** | 0.03 | (-0.22, 0.29) | 0.04 | (-0.21, 0.29) |
| Widowed | **-0.49** | **(-0.70, -0.29)** | **-0.24** | **(-0.46, -0.03)** | -0.20 | (-0.41, 0.01) |
| Never married | -0.02 | (-0.54, 0.49) | 0.30 | (-0.22, 0.82) | 0.24 | (-0.26, 0.74) |
| Age (50-59 ref category) |  |  |  |  |  |  |
| 60-69 | **-1.29** | **(-1.52, -1.07)** | **-1.33** | **(-1.56, -1.10)** | **-1.22** | **(-1.45, -1.00)** |
| 70-79 | **-2.69** | **(-2.95, -2.44)** | **-2.75** | **(-3.00, -2.50)** | **-2.41** | **(-2.66, -2.15)** |
| 80+ | **-5.00** | **(-5.30, -4.69)** | **-5.05** | **(-5.36, -4.75)** | **-4.27** | **(-4.58, -3.95)** |
| Ethnicity (white ref category) |  |  |  |  |  |  |
| Hispanic | **-0.76** | **(-1.04, -0.48)** | **-0.50** | **(-0.78, -0.21)** | -0.28 | (-0.56, 0.00) |
| Black | 0.93 | (0.70, 1.16) | 1.19 | (0.96, 1.42) | 1.27 | (1.05, 1.50) |
| Other | 0.14 | (-0.52, 0.79) | 0.19 | (-0.46, 0.84) | 0.48 | (-0.15, 1.11) |
| Work status (working ref category) |  |  |  |  |  |  |
| Not working | **-0.93** | **(-1.13, -0.74)** | **-0.86** | **(-1.06, -0.67)** | **-0.45** | **(-0.65, -0.26)** |
| Parental status (has children ref category) |  |  |  |  |  |  |
| No children | -0.28 | (-0.65, 0.09) | -0.33 | (-0.70, 0.04) | -0.29 | (-0.64, 0.07) |
| Education (low ref category) |  |  |  |  |  |  |
| Medium |  |  | 0.14 | (-0.06, 0.34) | -0.01 | (-0.20, 0.19) |
| High |  |  | 0.12 | (-0.11, 0.34) | -0.02 | (-0.24, 0.19) |
| Wealth (lowest wealth quintile ref category) |  |  |  |  |  |  |
| 2nd |  |  | **0.74** | **(0.50, 0.98)** | **0.54** | **(0.30, 0.77)** |
| 3rd |  |  | **0.93** | **(0.67, 1.18)** | **0.56** | **(0.31, 0.81)** |
| 4th |  |  | **1.07** | **(0.80, 1.33)** | **0.70** | **(0.44, 0.96)** |
| 5th high wealth |  |  | **1.07** | **(0.79, 1.35)** | **0.65** | **(0.37, 0.93)** |
| Smoking status (never smoked ref category) |  |  |  |  |  |  |
| Former smoker |  |  |  |  | 0.13 | (-0.04, 0.29) |
| Current smoker |  |  |  |  | **0.60** | **(0.37, 0.84)** |
| Physical activity (moderate activity ref category) |  |  |  |  |  |  |
| Sedentary |  |  |  |  | -1.90 | (-2.27, -1.53) |
| Low |  |  |  |  | -0.45 | (-0.63, -0.26) |
| High |  |  |  |  | 0.10 | (-0.10, 0.29) |
| Body Mass Index (BMI<25 ref category) |  |  |  |  |  |  |
| Overweight BMI (25 -29) |  |  |  |  | **0.60** | **(0.42, 0.79)** |
| Obese BMI (30+) |  |  |  |  | **1.18** | **(0.98, 1.37)** |
| Self-rated health (excellent / v. good ref category) |  |  |  |  |  |  |
| Good |  |  |  |  | **-0.28** | **(-0.46, -0.09)** |
| Fair / poor |  |  |  |  | **-0.98** | **(-1.21, -0.76)** |
| Chronic health conditions (0 reported conditions ref category) |  |  |  |  |  |  |
| Reported 1 condition |  |  |  |  | **-0.46** | **(-0.73, -0.19)** |
| Reported 2 conditions |  |  |  |  | **-0.59** | **(-0.86, -0.31)** |
| Reported 3+ conditions |  |  |  |  | **-1.16** | **(-1.45, -0.87)** |
| CES-D (CES-D<3 ref category) |  |  |  |  |  |  |
| CES-D≥3 |  |  |  |  | **-0.37** | **(-0.56, -0.18)** |
| *Constant* | *17.93* | *(17.71, 18.15)* | *16.92* | *(16.62, 17.23)* | *17.24* | *(16.85, 17.63)* |

*Results p<0.05 are shown in bold.*

*Model 1: Age and demographics (ethnicity, work status and parental status)*

*Model 2: Age + demographic and socio-economic measures (education and wealth)*

*Model 3: Age + demographic and socio-economic measures + health behaviours (smoking status, physical activity and BMI) + physical health and psychological morbidity (self-rated health, chronic health conditions and psychological morbidity)*

### Walking Speed

S3E Table: Walking speed models with coefficients for all covariates, men in ELSA

|  | Model1 | | Model 2 | | Model 3 | |
| --- | --- | --- | --- | --- | --- | --- |
|  | **Coef** | **95% CI** | **Coef** | **95% CI** | **Coef** | **95% CI** |
| Marital status (first marriage ref category) |  |  |  |  |  |  |
| Remarried | -0.002 | (-0.041, 0.037) | 0.011 | (-0.026, 0.048) | 0.014 | (-0.020, 0.048) |
| Divorced / separated | **-0.086** | **(-0.138, -0.034)** | -0.033 | (-0.083, 0.018) | -0.015 | (-0.061, 0.032) |
| Widowed | **-0.080** | **(-0.12, -0.041)** | **-0.046** | **(-0.085, -0.008)** | **-0.042** | **(-0.077, -0.006)** |
| Never married | **-0.113** | **(-0.187, -0.039)** | **-0.080** | **(-0.151, -0.010)** | **-0.082** | **(-0.147, -0.017)** |
| Age (60-69 ref category) |  |  |  |  |  |  |
| 70-79 | **-0.077** | **(-0.106, -0.047)** | **-0.068** | **(-0.096, -0.040)** | **-0.066** | **(-0.092, -0.040)** |
| 80+ | **-0.254** | **(-0.294, -0.213)** | **-0.245** | **(-0.284, -0.207)** | **-0.202** | **(-0.239, -0.165)** |
| Ethnicity (white ref category) |  |  |  |  |  |  |
| Non-white | **-0.233** | **(-0.325, -0.141)** | **-0.250** | **(-0.338, -0.161)** | **-0.179** | **(-0.261, -0.098)** |
| Work status (Working ref category) |  |  |  |  |  |  |
| Not working | **-0.067** | **(-0.109, -0.025)** | **-0.050** | **(-0.091, -0.010)** | -0.002 | (-0.040, 0.035) |
| Parental status (has children ref category) |  |  |  |  |  |  |
| No children | 0.009 | (-0.038, 0.055) | 0.004 | (-0.04, 0.049) | -0.002 | (-0.043, 0.038) |
| Education (low ref category) |  |  |  |  |  |  |
| Medium |  |  | **0.063** | **(0.034, 0.093)** | **0.044** | **(0.017, 0.071)** |
| High |  |  | **0.098** | **(0.058, 0.138)** | **0.065** | **(0.028, 0.102)** |
| Wealth (lowest wealth quintile ref category) |  |  |  |  |  |  |
| 2^nd^ |  |  | 0.036 | (-0.008, 0.08) | 0.015 | (-0.025, 0.056) |
| 3^rd^ |  |  | **0.081** | **(0.038, 0.124)** | 0.021 | (-0.018, 0.061) |
| 4^th^ |  |  | **0.121** | **(0.077, 0.164)** | **0.058** | **(0.018, 0.098)** |
| 5^th^ high wealth |  |  | **0.178** | **(0.133, 0.223)** | **0.094** | **(0.052, 0.137)** |
| Smoking status (never smoked ref category) |  |  |  |  |  |  |
| Former smoker |  |  |  |  | -0.003 | (-0.029, 0.023) |
| Current smoker |  |  |  |  | -0.023 | (-0.068, 0.022) |
| Physical activity (moderate activity ref category) |  |  |  |  |  |  |
| Sedentary |  |  |  |  | **-0.204** | **(-0.253, -0.155)** |
| Low |  |  |  |  | **-0.092** | **(-0.122, -0.061)** |
| High |  |  |  |  | **0.064** | **(0.034, 0.095)** |
| Body Mass Index (BMI<25 ref category) |  |  |  |  |  |  |
| Overweight BMI (25 -29) |  |  |  |  | 0.000 | (-0.029, 0.029) |
| Obese BMI (30+) |  |  |  |  | -0.030 | (-0.064, 0.004) |
| Self-rated health (excellent / v. good ref category) |  |  |  |  |  |  |
| Good |  |  |  |  | **-0.042** | **(-0.069, -0.014)** |
| Fair / poor |  |  |  |  | **-0.121** | **(-0.154, -0.087)** |
| Chronic health conditions (0 reported conditions ref category) |  |  |  |  |  |  |
| Reported 1 condition |  |  |  |  | 0.004 | (-0.028, 0.036) |
| Reported 2 conditions |  |  |  |  | -0.001 | (-0.035, 0.033) |
| Reported 3+ conditions |  |  |  |  | **-0.045** | **(-0.083, -0.007)** |
| CES-D (CES-D<3 ref category) |  |  |  |  |  |  |
| CES-D≥3 |  |  |  |  | -0.031 | (-0.066, 0.004) |
| *Constant* | *1.030* | *(0.987, 1.072)* | *0.871* | *(0.818, 0.924)* | *0.972* | *(0.910, 1.034)* |

*Results p<0.05 are shown in bold.*

*Model 1: Age and demographics (ethnicity, work status and parental status)*

*Model 2: Age + demographic and socio-economic measures (education and wealth)*

*Model 3: Age + demographic and socio-economic measures + health behaviours (smoking status, physical activity and BMI) + physical health and psychological morbidity (self-rated health, chronic health conditions and psychological morbidity)*

S3F Table: Walking speed models with coefficients for all covariates, men in HRS

|  | Model1 | | Model 2 | | Model 3 | |
| --- | --- | --- | --- | --- | --- | --- |
|  | **Coef** | **95% CI** | **Coef** | **95% CI** | **Coef** | **95% CI** |
| Marital status (first marriage ref category) |  |  |  |  |  |  |
| Remarried | 0.004 | (-0.016, 0.024) | 0.012 | (-0.008, 0.032) | 0.016 | (-0.003, 0.035) |
| Divorced / separated | **-0.034** | **(-0.064, -0.003)** | -0.001 | (-0.032, 0.029) | -0.002 | (-0.031, 0.027) |
| Widowed | **-0.068** | **(-0.094, -0.041)** | **-0.043** | **(-0.07, -0.017)** | **-0.037** | **(-0.062, -0.011)** |
| Never married | -0.035 | (-0.101, 0.032) | -0.010 | (-0.075, 0.056) | -0.021 | (-0.083, 0.041) |
| Age (65-69 ref category) |  |  |  |  |  |  |
| 70-79 | **-0.059** | **(-0.079, -0.040)** | **-0.061** | **(-0.080, -0.042)** | **-0.055** | **(-0.073, -0.036)** |
| 80+ | **-0.176** | **(-0.20, -0.151)** | **-0.182** | **(-0.206, -0.159)** | **-0.152** | **(-0.175, -0.128)** |
| Ethnicity (white ref category) |  |  |  |  |  |  |
| Hispanic | **-0.115** | **(-0.147, -0.084)** | **-0.071** | **(-0.102, -0.039)** | **-0.065** | **(-0.096, -0.035)** |
| Black | **-0.171** | **(-0.198, -0.144)** | **-0.125** | **(-0.153, -0.098)** | **-0.116** | **(-0.142, -0.09)** |
| Other | -0.031 | (-0.106, 0.044) | -0.030 | (-0.104, 0.044) | -0.044 | (-0.114, 0.027) |
| Work status (working ref category) |  |  |  |  |  |  |
| Not working | **-0.077** | **(-0.098, -0.055)** | **-0.063** | **(-0.084, -0.042)** | **-0.031** | **(-0.051, -0.010)** |
| Parental status (has children ref category) |  |  |  |  |  |  |
| No children | -0.039 | (-0.084, 0.006) | **-0.049** | **(-0.093, -0.005)** | -0.039 | (-0.08, 0.003) |
| Education (low ref category) |  |  |  |  |  |  |
| Medium |  |  | **0.050** | **(0.028, 0.072)** | **0.043** | **(0.022, 0.064)** |
| High |  |  | **0.053** | **(0.033, 0.074)** | **0.036** | **(0.016, 0.056)** |
| Wealth (lowest wealth quintile ref category) |  |  |  |  |  |  |
| 2^nd^ |  |  | **0.035** | **(0.005, 0.065)** | 0.014 | (-0.015, 0.042) |
| 3^rd^ |  |  | **0.065** | **(0.036, 0.095)** | **0.028** | **(0.000, 0.056)** |
| 4^th^ |  |  | **0.110** | **(0.081, 0.140)** | **0.059** | **(0.031, 0.087)** |
| 5^th^ high wealth |  |  | **0.123** | **(0.093, 0.154)** | **0.064** | **(0.035, 0.093)** |
| Smoking status (never smoked ref category) |  |  |  |  |  |  |
| Former smoker |  |  |  |  | -0.001 | (-0.018, 0.016) |
| Current smoker |  |  |  |  | -0.019 | (-0.046, 0.009) |
| Physical activity (moderate activity ref category) |  |  |  |  |  |  |
| Sedentary |  |  |  |  | **-0.178** | **(-0.210, -0.147)** |
| Low |  |  |  |  | **-0.051** | **(-0.072, -0.031)** |
| High |  |  |  |  | **0.029** | **(0.011, 0.048)** |
| Body Mass Index (BMI<25 ref category) |  |  |  |  |  |  |
| Overweight BMI (25 -29) |  |  |  |  | 0.010 | (-0.008, 0.029) |
| Obese BMI (30+) |  |  |  |  | -0.004 | (-0.025, 0.016) |
| Self-rated health (excellent / v. good ref category) |  |  |  |  |  |  |
| Good |  |  |  |  | **-0.025** | **(-0.044, -0.007)** |
| Fair / poor |  |  |  |  | **-0.079** | **(-0.101, -0.058)** |
| Chronic health conditions (0 reported conditions (ref category) |  |  |  |  |  |  |
| Reported 1 condition |  |  |  |  | -0.014 | (-0.046, 0.017) |
| Reported 2 conditions |  |  |  |  | -0.023 | (-0.053, 0.007) |
| Reported 3+ conditions |  |  |  |  | **-0.054** | **(-0.084, -0.024)** |
| CES-D (CES-D<3 ref category) |  |  |  |  |  |  |
| CES-D≥3 |  |  |  |  | **-0.027** | **(-0.050, -0.005)** |
| *Constant* | *0.952* | *(0.929, 0.974)* | *0.828* | *(0.794, 0.862)* | *0.918* | *(0.873, 0.963)* |

*Results p<0.05 are shown in bold.*

*Model 1: Age and demographics (ethnicity, work status and parental status)*

*Model 2: Age + demographic and socio-economic measures (education and wealth)*

*Model 3: Age + demographic and socio-economic measures + health behaviours (smoking status, physical activity and BMI) + physical health and psychological morbidity (self-rated health, chronic health conditions and psychological morbidity)*

S3G Table: Walking speed models with coefficients for all covariates, women in ELSA

|  | Model1 | | Model 2 | | Model 3 | |
| --- | --- | --- | --- | --- | --- | --- |
|  | **Coef** | **95% CI** | **Coef** | **95% CI** | **Coef** | **95% CI** |
| Marital status (first marriage ref category) |  |  |  |  |  |  |
| Remarried | -0.032 | (-0.075, 0.012) | -0.009 | (-0.051, 0.033) | 0.020 | (-0.017, 0.056) |
| Divorced / separated | **-0.057** | **(-0.096, -0.018)** | 0.004 | (-0.035, 0.042) | -0.002 | (-0.036, 0.032) |
| Widowed | **-0.057** | **(-0.085, -0.030)** | -0.013 | (-0.040, 0.014) | 0.000 | (-0.024, 0.024) |
| Never married | **-0.075** | **(-0.140, -0.011)** | -0.046 | (-0.108, 0.016) | -0.033 | (-0.087, 0.022) |
| Age (60-69 ref category) |  |  |  |  |  |  |
| 70-79 | **-0.107** | **(-0.133, -0.08)** | **-0.093** | **(-0.119, -0.068)** | **-0.069** | **(-0.092, -0.047)** |
| 80+ | **-0.299** | **(-0.335, -0.264)** | **-0.282** | **(-0.316, -0.248)** | **-0.220** | **(-0.251, -0.189)** |
| Ethnicity (white ref category) |  |  |  |  |  |  |
| Non-white | **-0.229** | **(-0.319, -0.139)** | **-0.214** | **(-0.299, -0.128)** | **-0.148** | **(-0.223, -0.072)** |
| Work status (Working ref category) |  |  |  |  |  |  |
| Not working | **-0.127** | **(-0.174, -0.081)** | **-0.105** | **(-0.150, -0.061)** | **-0.050** | **(-0.089, -0.010)** |
| Parental status (has children ref category) |  |  |  |  |  |  |
| No children | 0.015 | (-0.023, 0.053) | -0.001 | (-0.037, 0.035) | -0.002 | (-0.034, 0.029) |
| Education (low ref category) |  |  |  |  |  |  |
| Medium |  |  | **0.059** | **(0.035, 0.083)** | **0.028** | **(0.006, 0.049)** |
| High |  |  | **0.100** | **(0.061, 0.139)** | **0.053** | **(0.019, 0.088)** |
| Wealth (lowest wealth quintile ref category) |  |  |  |  |  |  |
| 2^nd^ |  |  | 0.033 | (-0.002, 0.068) | 0.007 | (-0.023, 0.038) |
| 3^rd^ |  |  | **0.100** | **(0.065, 0.135)** | **0.046** | **(0.015, 0.077)** |
| 4^th^ |  |  | **0.147** | **(0.111, 0.183)** | **0.067** | **(0.034, 0.099)** |
| 5^th^ high wealth |  |  | **0.192** | **(0.153, 0.232)** | **0.103** | **(0.068, 0.139)** |
| Smoking status (never smoked ref category) |  |  |  |  |  |  |
| Former smoker |  |  |  |  | 0.004 | (-0.016, 0.024) |
| Current smoker |  |  |  |  | 0.013 | (-0.022, 0.048) |
| Physical activity (moderate activity ref category) |  |  |  |  |  |  |
| Sedentary |  |  |  |  | **-0.196** | **(-0.233, -0.159)** |
| Low |  |  |  |  | **-0.107** | **(-0.129, -0.084)** |
| High |  |  |  |  | 0.009 | (-0.022, 0.040) |
| Body Mass Index (BMI<25 ref category) |  |  |  |  |  |  |
| Overweight BMI (25 -29) |  |  |  |  | **-0.026** | **(-0.049, -0.002)** |
| Obese BMI (30+) |  |  |  |  | **-0.058** | **(-0.083, -0.033)** |
| Self-rated health (excellent / v. good ref category) |  |  |  |  |  |  |
| Good |  |  |  |  | **-0.060** | **(-0.084, -0.036)** |
| Fair / poor |  |  |  |  | **-0.167** | **(-0.195, -0.139)** |
| Chronic health conditions (0 reported conditions ref category) |  |  |  |  |  |  |
| Reported 1 condition |  |  |  |  | -0.007 | (-0.036, 0.022) |
| Reported 2 conditions |  |  |  |  | -0.021 | (-0.051, 0.009) |
| Reported 3+ conditions |  |  |  |  | **-0.061** | **(-0.094, -0.029)** |
| CES-D (CES-D<3 ref category) |  |  |  |  |  |  |
| CES-D≥3 |  |  |  |  | **-0.045** | **(-0.067, -0.022)** |
| *Constant* | *1.042* | *(0.995, 1.09)* | *0.862* | *(0.808, 0.916)* | *1.027* | *(0.972, 1.081)* |

*Results p<0.05 are shown in bold.*

*Model 1: Age and demographics (ethnicity, work status and parental status)*

*Model 2: Age + demographic and socio-economic measures (education and wealth)*

*Model 3: Age + demographic and socio-economic measures + health behaviours (smoking status, physical activity and BMI) + physical health and psychological morbidity (self-rated health, chronic health conditions and psychological morbidity)*

S3H Table: Walking speed models with coefficients for all covariates, women in HRS

|  | Model1 | | Model 2 | | Model 3 | |
| --- | --- | --- | --- | --- | --- | --- |
| walk | **Coef** | **95% CI** | **Coef** | **95% CI** | **Coef** | **95% CI** |
| Marital status (first marriage ref category) |  |  |  |  |  |  |
| Remarried | 0.000 | (-0.023, 0.023) | 0.013 | (-0.009, 0.035) | 0.015 | (-0.006, 0.036) |
| Divorced / separated | **-0.046** | **(-0.070, -0.023)** | -0.001 | (-0.025, 0.023) | 0.001 | (-0.022, 0.023) |
| Widowed | **-0.051** | **(-0.068, -0.034)** | -0.014 | (-0.031, 0.003) | -0.008 | (-0.024, 0.007) |
| Never married | -0.043 | (-0.095, 0.008) | -0.015 | (-0.065, 0.035) | -0.028 | (-0.074, 0.019) |
| Age (65-69 ref category) |  |  |  |  |  |  |
| 70-79 | **-0.085** | **(-0.102, -0.069)** | **-0.085** | **(-0.101, -0.069)** | **-0.077** | **(-0.092, -0.062)** |
| 80+ | **-0.228** | **(-0.248, -0.207)** | **-0.228** | **(-0.248, -0.209)** | **-0.204** | **(-0.223, -0.185)** |
| Ethnicity (white ref category) |  |  |  |  |  |  |
| Hispanic | **-0.122** | **(-0.147, -0.096)** | **-0.076** | **(-0.101, -0.051)** | **-0.049** | **(-0.073, -0.026)** |
| Black | **-0.155** | **(-0.175, -0.134)** | **-0.113** | **(-0.133, -0.092)** | **-0.084** | **(-0.103, -0.065)** |
| Other | 0.020 | (-0.044, 0.085) | 0.025 | (-0.038, 0.087) | 0.024 | (-0.034, 0.082) |
| Work status (working ref category) |  |  |  |  |  |  |
| Not working | **-0.083** | **(-0.103, -0.062)** | **-0.072** | **(-0.092, -0.052)** | **-0.034** | **(-0.053, -0.015)** |
| Parental status (has children ref category) |  |  |  |  |  |  |
| No children | 0.003 | (-0.031, 0.037) | -0.002 | (-0.035, 0.031) | 0.003 | (-0.028, 0.033) |
| Education (low ref category) |  |  |  |  |  |  |
| Medium |  |  | 0.052 | (0.035, 0.069) | 0.035 | (0.019, 0.051) |
| High |  |  | 0.050 | (0.030, 0.070) | 0.025 | (0.006, 0.043) |
| Wealth (lowest wealth quintile ref category) |  |  |  |  |  |  |
| 2^nd^ |  |  | **0.047** | **(0.026, 0.068)** | **0.024** | **(0.004, 0.043)** |
| 3^rd^ |  |  | **0.100** | **(0.078, 0.121)** | **0.055** | **(0.035, 0.076)** |
| 4^th^ |  |  | **0.132** | **(0.110, 0.155)** | **0.077** | **(0.056, 0.099)** |
| 5^th^ high wealth |  |  | **0.137** | **(0.113, 0.161)** | **0.072** | **(0.049, 0.094)** |
| Smoking status (never smoked ref category) |  |  |  |  |  |  |
| Former smoker |  |  |  |  | 0.006 | (-0.007, 0.020) |
| Current smoker |  |  |  |  | 0.013 | (-0.008, 0.034) |
| Physical activity (moderate activity ref category) |  |  |  |  |  |  |
| Sedentary |  |  |  |  | **-0.189** | **(-0.216, -0.162)** |
| Low |  |  |  |  | **-0.062** | **(-0.077, -0.047)** |
| High |  |  |  |  | 0.000 | (-0.017, 0.018) |
| Body Mass Index (BMI<25 ref category) |  |  |  |  |  |  |
| Overweight BMI (25 -29) |  |  |  |  | 0.002 | (-0.013, 0.017) |
| Obese BMI (30+) |  |  |  |  | **-0.038** | **(-0.054, -0.021)** |
| Self-rated health (excellent / v. good ref category) |  |  |  |  |  |  |
| Good |  |  |  |  | **-0.045** | **(-0.060, -0.030)** |
| Fair / poor |  |  |  |  | **-0.101** | **(-0.119, -0.082)** |
| Chronic health conditions (0 reported conditions (ref category) |  |  |  |  |  |  |
| Reported 1 condition |  |  |  |  | -0.004 | (-0.032, 0.024) |
| Reported 2 conditions |  |  |  |  | -0.016 | (-0.044, 0.011) |
| Reported 3+ conditions |  |  |  |  | **-0.048** | **(-0.076, -0.020)** |
| CES-D (CES-D<3 ref category) |  |  |  |  |  |  |
| CES-D≥3 |  |  |  |  | **-0.034** | **(-0.049, -0.018)** |
| *Constant* | *0.921* | *(0.899, 0.943)* | *0.781* | *(0.753, 0.81)* | *0.893* | *(0.856, 0.931)* |

*Results p<0.05 are shown in bold.*

*Model 1: Age and demographics (ethnicity, work status and parental status)*

*Model 2: Age + demographic and socio-economic measures (education and wealth)*

*Model 3: Age + demographic and socio-economic measures + health behaviours (smoking status, physical activity and BMI) + physical health and psychological morbidity (self-rated health, chronic health conditions and psychological morbidity)*

## Models with each covariate individually

S3I Table: Grip strength and marital status for men and women in ELSA regressed on each coefficient individually

|  | Men | | Women | |
| --- | --- | --- | --- | --- |
|  | **Coef.** | **95% CI** | **Coef.** | **95% CI** |
| Marital status (first marriage ref category) |  |  |  |  |
| Remarried | **0.61** | **(0.14, 1.07)** | 0.18 | (-0.17, 0.52) |
| Divorced / separated | -0.27 | (-0.81, 0.27) | **-0.39** | **(-0.71, -0.07)** |
| Widowed | **-0.89** | **(-1.53, -0.26)** | **-0.33** | **(-0.64, -0.03)** |
| Never married | **-1.46** | **(-2.12, -0.81)** | -0.36 | (-0.85, 0.13) |
| Age (50-59 ref category) |  |  |  |  |
| 60-69 | **-2.11** | **(-2.50, -1.71)** | **-1.15** | **(-1.41, -0.88)** |
| 70-79 | **-4.82** | **(-5.27, -4.37)** | **-3.03** | **(-3.33, -2.72)** |
| 80+ | **-8.19** | **(-8.85, -7.53)** | **-5.48** | **(-5.91, -5.05)** |
| *Constant* | *25.74* | *(25.41, 26.06)* | *16.59* | *(16.36, 16.81)* |

|  | Men | | Women | |
| --- | --- | --- | --- | --- |
|  | **Coef.** | **95% CI** | **Coef.** | **95% CI** |
| Marital status (first marriage ref category) |  |  |  |  |
| Remarried | **0.61** | **(0.14, 1.07)** | 0.17 | (-0.17, 0.51) |
| Divorced / separated | -0.28 | (-0.82, 0.26) | **-0.39** | **(-0.71, -0.07)** |
| Widowed | **-0.92** | **(-1.55, -0.29)** | **-0.33** | **(-0.64, -0.02)** |
| Never married | **-1.51** | **(-2.17, -0.86)** | -0.35 | (-0.85, 0.14) |
| Ethnicity (white ref category) |  |  |  |  |
| Non-white | **-2.31** | **(-3.26, -1.36)** | -0.43 | (-1.12, 0.26) |
| *Constant* | *25.84* | *(25.51, 26.17)* | *16.61* | *(16.38, 16.83)* |

*Adjusted for age*

|  | Men | | Women | |
| --- | --- | --- | --- | --- |
|  | **Coef.** | **95% CI** | **Coef.** | **95% CI** |
| Marital status (first marriage ref category) |  |  |  |  |
| Remarried | **0.61** | **(0.14, 1.07)** | 0.14 | (-0.20, 0.49) |
| Divorced / separated | -0.15 | (-0.69, 0.39) | **-0.42** | **(-0.73, -0.10)** |
| Widowed | **-0.72** | **(-1.35, -0.10)** | -0.30 | (-0.61, 0.00) |
| Never married | **-1.24** | **(-1.90, -0.59)** | -0.35 | (-0.84, 0.14) |
| Work status (working ref category) |  |  |  |  |
| Not working | **-1.73** | **(-2.13, -1.34)** | **-0.90** | **(-1.18, -0.62)** |
| *Constant* | *26.05* | *(25.72, 26.39)* | *16.86* | *(16.62, 17.1)* |

*Adjusted for age*

|  | Men | | Women | |
| --- | --- | --- | --- | --- |
|  | **Coef.** | **95% CI** | **Coef.** | **95% CI** |
| Marital status (first marriage ref category) |  |  |  |  |
| Remarried | **0.61** | **(0.15, 1.08)** | 0.18 | (-0.17, 0.52) |
| Divorced / separated | -0.21 | (-0.75, 0.34) | **-0.40** | **(-0.72, -0.08)** |
| Widowed | **-0.88** | **(-1.51, -0.25)** | **-0.34** | **(-0.65, -0.03)** |
| Never married | **-0.80** | **(-1.57, -0.03)** | -0.48 | (-1.02, 0.07) |
| Parental status (has children ref category) |  |  |  |  |
| No children | **-0.85** | **(-1.36, -0.33)** | 0.16 | (-0.17, 0.49) |
| *Constant* | *25.83* | *(25.49, 26.16)* | *16.57* | *(16.35, 16.8)* |

*Adjusted for age*

|  | Men | | Women | |
| --- | --- | --- | --- | --- |
|  | **Coef.** | **95% CI** | **Coef.** | **95% CI** |
| Marital status (first marriage ref category) |  |  |  |  |
| Remarried | **0.61** | **(0.14, 1.08)** | 0.27 | (-0.08, 0.61) |
| Divorced / separated | -0.22 | (-0.76, 0.33) | **-0.34** | **(-0.66, -0.02)** |
| Widowed | **-0.85** | **(-1.48, -0.21)** | -0.27 | (-0.58, 0.03) |
| Never married | **-1.46** | **(-2.12, -0.80)** | **-0.52** | **(-1.01, -0.02)** |
| Education (low ref category) |  |  |  |  |
| Medium | **0.48** | **(0.12, 0.84)** | **0.49** | **(0.26, 0.73)** |
| High | 0.24 | (-0.20, 0.69) | **1.07** | **(0.75, 1.40)** |
| *Constant* | *25.47* | *(25.06, 25.87)* | *16.10* | *(15.83, 16.37)* |

*Adjusted for age*

|  | Men | | Women | |
| --- | --- | --- | --- | --- |
|  | **Coef.** | **95% CI** | **Coef.** | **95% CI** |
| Marital status (first marriage ref category) |  |  |  |  |
| Remarried | **0.79** | **(0.32, 1.25)** | 0.30 | (-0.04, 0.64) |
| Divorced / separated | 0.39 | (-0.17, 0.95) | 0.02 | (-0.31, 0.35) |
| Widowed | -0.55 | (-1.18, 0.08) | -0.09 | (-0.4, 0.22) |
| Never married | **-1.06** | **(-1.72, -0.40)** | -0.12 | (-0.62, 0.37) |
| Wealth (lowest wealth quintile ref category) |  |  |  |  |
| 2^nd^ | **0.84** | **(0.27, 1.41)** | 0.25 | (-0.10, 0.60) |
| 3^rd^ | **1.74** | **(1.18, 2.31)** | **0.69** | **(0.34, 1.04)** |
| 4^th^ | **1.99** | **(1.44, 2.54)** | **1.01** | **(0.66, 1.37)** |
| 5^th^ high wealth | **2.07** | **(1.52, 2.62)** | 1.35 | (0.99, 1.71) |
| *Constant* | *24.13* | *(23.59, 24.68)* | *15.76* | *(15.4, 16.11)* |

*Adjusted for age*

|  | Men | | Women | |
| --- | --- | --- | --- | --- |
|  | **Coef.** | **95% CI** | **Coef.** | **95% CI** |
| Marital status (first marriage ref category) |  |  |  |  |
| Remarried | **0.65** | **(0.18, 1.12)** | 0.17 | (-0.18, 0.51) |
| Divorced / separated | -0.06 | (-0.61, 0.48) | **-0.41** | **(-0.73, -0.08)** |
| Widowed | **-0.81** | **(-1.44, -0.18)** | **-0.34** | **(-0.65, -0.03)** |
| Never married | **-1.32** | **(-1.98, -0.66)** | -0.37 | (-0.86, 0.13) |
| Smoking status (never smoked ref category) |  |  |  |  |
| Former smoker | **0.13** | **(-0.22, 0.49)** | 0.06 | (-0.17, 0.29) |
| Current smoker | **-1.10** | **(-1.63, -0.56)** | 0.08 | (-0.25, 0.42) |
| *Constant* | *25.82* | *(25.44, 26.2)* | *16.56* | *(16.31, 16.81)* |

*Adjusted for age*

|  | Men | | Women | |
| --- | --- | --- | --- | --- |
|  | **Coef.** | **95% CI** | **Coef.** | **95% CI** |
| Marital status (first marriage ref category) |  |  |  |  |
| Remarried | **0.64** | **(0.19, 1.10)** | 0.23 | (-0.10, 0.57) |
| Divorced / separated | -0.11 | (-0.64, 0.43) | -0.27 | (-0.58, 0.04) |
| Widowed | **-0.72** | **(-1.34, -0.10)** | -0.23 | (-0.53, 0.07) |
| Never married | **-1.28** | **(-1.93, -0.63)** | -0.27 | (-0.75, 0.21) |
| Physical activity (moderate activity ref category) |  |  |  |  |
| Sedentary | **-2.91** | **(-3.66, -2.15)** | **-2.22** | **(-2.71, -1.73)** |
| Low | **-1.23** | **(-1.67, -0.80)** | **-0.96** | **(-1.21, -0.71)** |
| High | **0.88** | **(0.50, 1.26)** | 0.80 | (0.51, 1.09) |
| *Constant* | *25.64* | *(25.28, 25.99)* | *16.63* | *(16.39, 16.87)* |

*Adjusted for age*

|  | Men | | Women | |
| --- | --- | --- | --- | --- |
|  | **Coef.** | **95% CI** | **Coef.** | **95% CI** |
| Marital status (first marriage ref category) |  |  |  |  |
| Remarried | **0.57** | **(0.11, 1.04)** | 0.17 | (-0.17, 0.52) |
| Divorced / separated | -0.23 | (-0.77, 0.31) | -0.38 | (-0.70, -0.06) |
| Widowed | **-0.91** | **(-1.54, -0.28)** | **-0.33** | **(-0.63, -0.02)** |
| Never married | **-1.28** | **(-1.94, -0.62)** | -0.35 | (-0.84, 0.15) |
| Body Mass Index (BMI<25 ref category) |  |  |  |  |
| Overweight BMI (25 -29) | **1.26** | **(0.85, 1.67)** | 0.25 | (-0.01, 0.51) |
| Obese BMI (30+) | **1.41** | **(0.96, 1.86)** | 0.17 | (-0.10, 0.44) |
| *Constant* | *24.69* | *(24.24, 25.14)* | *16.45* | *(16.18, 16.72)* |

*Adjusted for age*

|  | Men | | Women | |
| --- | --- | --- | --- | --- |
|  | **Coef.** | **95% CI** | **Coef.** | **95% CI** |
| Marital status (first marriage ref category) |  |  |  |  |
| Remarried | **0.64** | **(0.19, 1.10)** | 0.32 | (-0.02, 0.65) |
| Divorced / separated | 0.01 | (-0.53, 0.54) | -0.13 | (-0.45, 0.18) |
| Widowed | **-0.81** | **(-1.43, -0.19)** | -0.20 | (-0.50, 0.10) |
| Never married | **-1.32** | **(-1.96, -0.67)** | -0.27 | (-0.76, 0.21) |
| Self-rated health (excellent / v. good ref category) |  |  |  |  |
| Good | **-0.55** | **(-0.92, -0.18)** | **-0.85** | **(-1.09, -0.61)** |
| Fair / poor | **-2.28** | **(-2.68, -1.88)** | **-1.95** | **(-2.22, -1.69)** |
| *Constant* | *26.29* | *(25.94, 26.65)* | *17.14* | *(16.9, 17.38)* |

*Adjusted for age*

|  | Men | | Women | |
| --- | --- | --- | --- | --- |
|  | **Coef.** | **95% CI** | **Coef.** | **95% CI** |
| Marital status (first marriage ref category) |  |  |  |  |
| Remarried | **0.68** | **(0.21, 1.14)** | 0.25 | (-0.09, 0.59) |
| Divorced / separated | -0.20 | (-0.74, 0.34) | -0.31 | (-0.62, 0.01) |
| Widowed | **-0.81** | **(-1.43, -0.18)** | -0.22 | (-0.53, 0.08) |
| Never married | **-1.46** | **(-2.12, -0.81)** | -0.30 | (-0.79, 0.19) |
| Chronic health conditions (0 reported conditions ref category) |  |  |  |  |
| Reported 1 condition | -0.20 | (-0.60, 0.20) | **-0.32** | **(-0.59, -0.05)** |
| Reported 2 conditions | **-0.98** | **(-1.43, -0.53)** | **-0.92** | **(-1.22, -0.61)** |
| Reported 3+ conditions | **-1.71** | **(-2.24, -1.19)** | **-1.60** | **(-1.94, -1.25)** |
| *Constant* | *26.01* | *(25.64, 26.38)* | *16.88* | *(16.62, 17.14)* |

*Adjusted for age*

|  | Men | | Women | |
| --- | --- | --- | --- | --- |
|  | **Coef.** | **95% CI** | **Coef.** | **95% CI** |
| Marital status (first marriage ref category) |  |  |  |  |
| Remarried | **0.65** | **(0.19, 1.11)** | 0.30 | (-0.04, 0.64) |
| Divorced / separated | -0.09 | (-0.63, 0.46) | -0.21 | (-0.52, 0.11) |
| Widowed | **-0.68** | **(-1.31, -0.05)** | -0.12 | (-0.43, 0.18) |
| Never married | **-1.29** | **(-1.95, -0.64)** | -0.24 | (-0.73, 0.25) |
| CES-D (CES-D<3 ref category) |  |  |  |  |
| CES-D≥3 | **-1.50** | **(-1.96, -1.05)** | **-1.20** | **(-1.45, -0.96)** |
| *Constant* | *25.92* | *(25.59, 26.25)* | *16.80* | *(16.58, 17.03)* |

*Adjusted for age*

S3J Table: Grip strength and marital status for men and women in HRS regressed on each coefficient individually

|  | **Men** | | **Women** | |
| --- | --- | --- | --- | --- |
|  | Coef. | 95% CI | Coef. | 95% CI |
| **Marital status (first marriage ref category)** |  |  |  |  |
| Remarried | 0.26 | (-0.04, 0.55) | -0.06 | (-0.29, 0.18) |
| Divorced / separated | -0.79 | (-1.20, -0.38) | -0.10 | (-0.34, 0.14) |
| Widowed | -0.94 | (-1.40, -0.48) | -0.39 | (-0.60, -0.19) |
| Never married | -2.28 | (-3.00, -1.56) | 0.09 | (-0.38, 0.55) |
| **Age (50-59 ref category)** |  |  |  |  |
| 60-69 | -2.57 | (-2.91, -2.22) | -1.54 | (-1.77, -1.32) |
| 70-79 | -5.03 | (-5.38, -4.68) | -3.17 | (-3.40, -2.93) |
| 80+ | -8.54 | (-8.97, -8.10) | -5.61 | (-5.89, -5.32) |
| ***Constant*** | *26.74* | *(26.44, 27.04)* | *17.58* | *(17.38, 17.79)* |

*Adjusted for age*

|  | **Men** | | **Women** | |
| --- | --- | --- | --- | --- |
|  | Coef. | 95% CI | Coef. | 95% CI |
| **Marital status (first marriage ref category)** |  |  |  |  |
| Remarried | 0.22 | (-0.07, 0.52) | -0.09 | (-0.33, 0.14) |
| Divorced / separated | -0.72 | (-1.13, -0.31) | -0.25 | (-0.49, 0.00) |
| Widowed | -0.93 | (-1.39, -0.47) | -0.49 | (-0.70, -0.29) |
| Never married | -2.33 | (-3.05, -1.62) | -0.16 | (-0.62, 0.31) |
| **Ethnicity (white ref category)** |  |  |  |  |
| Hispanic | -1.73 | (-2.17, -1.28) | -0.86 | (-1.14, -0.58) |
| Black | -0.61 | (-1.00, -0.22) | 0.91 | (0.69, 1.14) |
| Other | -1.38 | (-2.41, -0.35) | 0.13 | (-0.52, 0.79) |
| ***Constant*** | *27.05* | *(26.74, 27.36)* | *17.59* | *(17.38, 17.8)* |

*Adjusted for age*

|  | **Men** | | **Women** | |
| --- | --- | --- | --- | --- |
|  | Coef. | 95% CI | Coef. | 95% CI |
| **Marital status (first marriage ref category**) |  |  |  |  |
| Remarried | 0.25 | (-0.04, 0.55) | -0.08 | (-0.31, 0.15) |
| Divorced / separated | -0.70 | (-1.10, -0.30) | -0.16 | (-0.40, 0.09) |
| Widowed | -0.85 | (-1.31, -0.40) | -0.39 | (-0.60, -0.19) |
| Never married | -2.12 | (-2.83, -1.41) | 0.06 | (-0.40, 0.53) |
| **Work status (working ref category)** |  |  |  |  |
| Not working | -1.51 | (-1.80, -1.21) | -0.96 | (-1.15, -0.76) |
| *Constant* | *27.07* | *(26.76, 27.37)* | *17.94* | *(17.72, 18.15)* |

*Adjusted for age*

|  | **Men** | | **Women** | |
| --- | --- | --- | --- | --- |
|  | Coef. | 95% CI | Coef. | 95% CI |
| **Marital status (first marriage ref category)** |  |  |  |  |
| Remarried | 0.25 | (-0.05, 0.55) | -0.06 | (-0.29, 0.18) |
| Divorced / separated | -0.76 | (-1.17, -0.36) | -0.09 | (-0.33, 0.16) |
| Widowed | -0.89 | (-1.36, -0.43) | -0.39 | (-0.60, -0.19) |
| Never married | -1.54 | (-2.37, -0.70) | 0.27 | (-0.24, 0.79) |
| **Parental status (has children ref category)** |  |  |  |  |
| No children | -1.05 | (-1.65, -0.44) | -0.32 | (-0.69, 0.05) |
| ***Constant*** | *26.79* | *(26.49, 27.1)* | *17.59* | *(17.39, 17.8)* |

*Adjusted for age*

|  | **Men** | | **Women** | |
| --- | --- | --- | --- | --- |
|  | Coef. | 95% CI | Coef. | 95% CI |
| **Marital status (first marriage ref category)** |  |  |  |  |
| Remarried | 0.25 | (-0.05, 0.54) | -0.04 | (-0.28, 0.19) |
| Divorced / separated | -0.76 | (-1.16, -0.35) | -0.08 | (-0.33, 0.16) |
| Widowed | -0.87 | (-1.33, -0.41) | -0.34 | (-0.55, -0.13) |
| Never married | -2.28 | (-3.00, -1.56) | 0.07 | (-0.40, 0.54) |
| **Education (low ref category)** |  |  |  |  |
| Medium | 0.75 | (0.43, 1.07) | 0.38 | (0.19, 0.58) |
| High | 0.53 | (0.24, 0.82) | 0.45 | (0.23, 0.66) |
| ***Constant*** | *26.34* | *(26, 26.68)* | *17.34* | *(17.11, 17.56)* |

*Adjusted for age*

|  | **Men** | | **Women** | |
| --- | --- | --- | --- | --- |
|  | Coef. | 95% CI | Coef. | 95% CI |
| **Marital status (first marriage ref category)** |  |  |  |  |
| Remarried | 0.39 | (0.09, 0.68) | -0.01 | (-0.24, 0.23) |
| Divorced / separated | -0.20 | (-0.62, 0.21) | 0.23 | (-0.03, 0.48) |
| Widowed | -0.55 | (-1.01, -0.09) | -0.16 | (-0.38, 0.05) |
| Never married | -1.85 | (-2.56, -1.13) | 0.41 | (-0.06, 0.89) |
| **Wealth (lowest wealth quintile ref category)** |  |  |  |  |
| 2nd | 0.93 | (0.51, 1.36) | 0.78 | (0.54, 1.03) |
| 3rd | 1.69 | (1.28, 2.11) | 0.88 | (0.63, 1.14) |
| 4th | 1.96 | (1.54, 2.37) | 0.97 | (0.72, 1.23) |
| 5th high wealth | 2.21 | (1.8, 2.62) | 0.98 | (0.72, 1.24) |
| ***Constant*** | *25.27* | *(24.85, 25.69)* | *16.78* | *(16.5, 17.05)* |

*Adjusted for age*

|  | **Men** | | **Women** | |
| --- | --- | --- | --- | --- |
|  | Coef. | 95% CI | Coef. | 95% CI |
| **Marital status (first marriage ref category)** |  |  |  |  |
| Remarried | 0.28 | (-0.01, 0.58) | -0.06 | (-0.30, 0.17) |
| Divorced / separated | -0.67 | (-1.08, -0.26) | -0.11 | (-0.36, 0.14) |
| Widowed | -0.87 | (-1.33, -0.41) | -0.40 | (-0.61, -0.19) |
| Never married | -2.27 | (-2.99, -1.55) | 0.08 | (-0.39, 0.55) |
| **Smoking status (never smoked ref category)** |  |  |  |  |
| Former smoker | -0.12 | (-0.39, 0.16) | 0.06 | (-0.12, 0.23) |
| Current smoker | -0.78 | (-1.16, -0.4) | 0.05 | (-0.19, 0.29) |
| ***Constant*** | *26.95* | *(26.62, 27.29)* | *17.56* | *(17.34, 17.77)* |

*Adjusted for age*

|  | **Men** | | **Women** | |
| --- | --- | --- | --- | --- |
|  | Coef. | 95% CI | Coef. | 95% CI |
| **Marital status (first marriage ref category)** |  |  |  |  |
| Remarried | 0.30 | (0.01, 0.59) | -0.04 | (-0.27, 0.19) |
| Divorced / separated | -0.65 | (-1.05, -0.25) | -0.03 | (-0.27, 0.21) |
| Widowed | -0.79 | (-1.24, -0.34) | -0.28 | (-0.48, -0.07) |
| Never married | -2.20 | (-2.90, -1.49) | 0.14 | (-0.32, 0.60) |
| **Physical activity (moderate activity ref category)** |  |  |  |  |
| Sedentary | -3.78 | (-4.31, -3.25) | -2.44 | (-2.81, -2.07) |
| Low | -1.18 | (-1.51, -0.86) | -0.65 | (-0.84, -0.47) |
| High | 0.35 | (0.07, 0.63) | 0.29 | (0.08, 0.49) |
| ***Constant*** | *26.83* | *(26.5, 27.16)* | *17.64* | *(17.42, 17.87)* |

*Adjusted for age*

|  | **Men** | | **Women** | |
| --- | --- | --- | --- | --- |
|  | Coef. | 95% CI | Coef. | 95% CI |
| **Marital status (first marriage ref category)** |  |  |  |  |
| Remarried | 0.23 | (-0.06, 0.52) | -0.07 | (-0.30, 0.17) |
| Divorced / separated | -0.66 | (-1.07, -0.26) | -0.14 | (-0.38, 0.11) |
| Widowed | -0.92 | (-1.38, -0.46) | -0.44 | (-0.64, -0.23) |
| Never married | -2.09 | (-2.80, -1.37) | 0.04 | (-0.42, 0.51) |
| **Body Mass Index (BMI<25 ref category)** |  |  |  |  |
| Overweight BMI (25 -29) | 1.16 | (0.85, 1.46) | 0.46 | (0.27, 0.65) |
| Obese BMI (30+) | 1.45 | (1.12, 1.77) | 0.66 | (0.47, 0.85) |
| ***Constant*** | *25.70* | *(25.33, 26.08)* | *17.19* | *(16.96, 17.42)* |

*Adjusted for age*

|  | **Men** | | **Women** | |
| --- | --- | --- | --- | --- |
|  | Coef. | 95% CI | Coef. | 95% CI |
| **Marital status (first marriage ref category)** |  |  |  |  |
| Remarried | 0.33 | (0.04, 0.62) | 0.01 | (-0.22, 0.24) |
| Divorced / separated | -0.56 | (-0.96, -0.16) | 0.10 | (-0.14, 0.34) |
| Widowed | -0.77 | (-1.22, -0.32) | -0.19 | (-0.40, 0.01) |
| Never married | -2.13 | (-2.84, -1.43) | 0.28 | (-0.18, 0.74) |
| **Self-rated health (excellent / v. good ref category)** |  |  |  |  |
| Good | -0.73 | (-1.01, -0.45) | -0.43 | (-0.61, -0.25) |
| Fair / poor | -2.34 | (-2.64, -2.05) | -1.66 | (-1.85, -1.47) |
| ***Constant*** | *27.43* | *(27.11, 27.74)* | *18.02* | *(17.8, 18.23)* |

*Adjusted for age*

|  | **Men** | | **Women** | |
| --- | --- | --- | --- | --- |
|  | Coef. | 95% CI | Coef. | 95% CI |
| **Marital status (first marriage ref category)** |  |  |  |  |
| Remarried | 0.32 | (0.03, 0.61) | -0.01 | (-0.24, 0.22) |
| Divorced / separated | -0.75 | (-1.15, -0.35) | 0.03 | (-0.21, 0.28) |
| Widowed | -0.86 | (-1.32, -0.41) | -0.28 | (-0.49, -0.08) |
| Never married | -2.32 | (-3.03, -1.61) | 0.16 | (-0.31, 0.62) |
| **Chronic health conditions (0 reported conditions ref category)** |  |  |  |  |
| Reported 1 condition | -0.30 | (-0.71, 0.10) | -0.49 | (-0.77, -0.21) |
| Reported 2 conditions | -1.17 | (-1.57, -0.77) | -0.70 | (-0.97, -0.42) |
| Reported 3+ conditions | -2.01 | (-2.41, -1.61) | -1.56 | (-1.84, -1.29) |
| ***Constant*** | *27.36* | *(26.97, 27.74)* | *18.12* | *(17.84, 18.39)* |

*Adjusted for age*

|  | **Men** | | **Women** | |
| --- | --- | --- | --- | --- |
|  | Coef. | 95% CI | Coef. | 95% CI |
| **Marital status (first marriage ref category)** |  |  |  |  |
| Remarried | 0.29 | (0.00, 0.59) | -0.05 | (-0.28, 0.18) |
| Divorced / separated | -0.62 | (-1.03, -0.22) | 0.03 | (-0.21, 0.28) |
| Widowed | -0.67 | (-1.13, -0.21) | -0.26 | (-0.47, -0.06) |
| Never married | -2.15 | (-2.87, -1.44) | 0.20 | (-0.26, 0.67) |
| **CES-D (CES-D<3 ref category)** |  |  |  |  |
| CES-D≥3 | -1.53 | (-1.87, -1.19) | -1.07 | (-1.26, -0.89) |
| ***Constant*** | *26.99* | *(26.69, 27.29)* | *17.81* | *(17.6, 18.01)* |

*Adjusted for age*

S3K Table: Walking speed and marital status for men and women in ELSA regressed on each coefficient individually

|  | **Men** | | **Women** | |
| --- | --- | --- | --- | --- |
|  | Coef. | 95% CI | Coef. | 95% CI |
| **Marital status (first marriage ref category)** |  |  |  |  |
| Remarried | -0.003 | (-0.043, 0.036) | -0.026 | (-0.071, 0.018) |
| Divorced / separated | -0.082 | (-0.134, -0.030) | -0.056 | (-0.096, -0.017) |
| Widowed | -0.080 | (-0.120, -0.040) | -0.057 | (-0.085, -0.029) |
| Never married | -0.103 | (-0.166, -0.041) | -0.064 | (-0.121, -0.007) |
| **Age (60-69 ref category)** |  |  |  |  |
| 70-79 | -0.087 | (-0.116, -0.057) | -0.118 | (-0.145, -0.091) |
| 80+ | -0.263 | (-0.304, -0.223) | -0.308 | (-0.344, -0.272) |
| ***Constant*** | *0.973* | *(0.948, 0.998)* | *0.928* | *(0.904, 0.952)* |

|  | **Men** | | **Women** | |
| --- | --- | --- | --- | --- |
| **Marital status (first marriage ref category)** | Coef. | 95% CI | Coef. | 95% CI |
| Remarried | 0.002 | (-0.037, 0.041) | -0.026 | (-0.07, 0.018) |
| Divorced / separated | -0.082 | (-0.134, -0.030) | -0.054 | (-0.093, -0.014) |
| Widowed | -0.082 | (-0.122, -0.043) | -0.057 | (-0.085, -0.029) |
| Never married | -0.105 | (-0.167, -0.043) | -0.062 | (-0.119, -0.005) |
| **Ethnicity (white ref category)** |  |  |  |  |
| Non-white | -0.235 | (-0.327, -0.143) | -0.227 | (-0.317, -0.136) |
| ***Constant*** | *0.976* | *(0.951, 1.001)* | *0.933* | *(0.909, 0.957)* |

*Adjusted for age*

|  | **Men** | | **Women** | |
| --- | --- | --- | --- | --- |
|  | Coef. | 95% CI | Coef. | 95% CI |
| **Marital status (first marriage ref category)** |  |  |  |  |
| Remarried | -0.007 | (-0.046, 0.032) | -0.031 | (-0.075, 0.013) |
| Divorced / separated | -0.085 | (-0.137, -0.032) | -0.059 | (-0.098, -0.019) |
| Widowed | -0.078 | (-0.118, -0.038) | -0.057 | (-0.084, -0.029) |
| Never married | -0.104 | (-0.166, -0.042) | -0.065 | (-0.122, -0.008) |
| **Work status (Working ref category)** |  |  |  |  |
| Not working | -0.068 | (-0.111, -0.026) | -0.127 | (-0.174, -0.08) |
| ***Constant*** | *1.028* | *(0.986, 1.071)* | *1.038* | *(0.991, 1.086)* |

*Adjusted for age*

|  | **Men** | | **Women** | |
| --- | --- | --- | --- | --- |
|  | Coef. | 95% CI | Coef. | 95% CI |
| **Marital status (first marriage ref category)** |  |  |  |  |
| Remarried | -0.004 | (-0.043, 0.036) | -0.027 | (-0.071, 0.018) |
| Divorced / separated | -0.083 | (-0.135, -0.030) | -0.056 | (-0.096, -0.017) |
| Widowed | -0.080 | (-0.121, -0.040) | -0.058 | (-0.086, -0.03) |
| Never married | -0.111 | (-0.186, -0.036) | -0.073 | (-0.138, -0.007) |
| **Parental status (has children ref category)** |  |  |  |  |
| No children | 0.009 | (-0.038, 0.056) | 0.011 | (-0.028, 0.049) |
| ***Constant*** | *0.972* | *(0.947, 0.997)* | *0.927* | *(0.903, 0.951)* |

*Adjusted for age*

|  | **Men** | | **Women** | |
| --- | --- | --- | --- | --- |
|  | Coef. | 95% CI | Coef. | 95% CI |
| **Marital status (first marriage ref category)** |  |  |  |  |
| Remarried | 0.002 | (-0.036, 0.041) | -0.020 | (-0.064, 0.023) |
| Divorced / separated | -0.073 | (-0.124, -0.022) | -0.056 | (-0.094, -0.017) |
| Widowed | -0.066 | (-0.105, -0.026) | -0.048 | (-0.076, -0.021) |
| Never married | -0.099 | (-0.160, -0.038) | -0.093 | (-0.149, -0.037) |
| **Education (low ref category)** |  |  |  |  |
| Medium | 0.105 | (0.077, 0.134) | 0.099 | (0.075, 0.123) |
| High | 0.151 | (0.113, 0.188) | 0.166 | (0.128, 0.205) |
| ***Constant*** | *0.909* | *(0.881, 0.937)* | *0.867* | *(0.841, 0.894)* |

*Adjusted for age*

|  | **Men** | | **Women** | |
| --- | --- | --- | --- | --- |
|  | Coef. | 95% CI | Coef. | 95% CI |
| **Marital status (first marriage ref category)** |  |  |  |  |
| Remarried | 0.007 | (-0.031, 0.044) | -0.005 | (-0.047, 0.038) |
| Divorced / separated | -0.022 | (-0.074, 0.029) | 0.017 | (-0.022, 0.056) |
| Widowed | -0.048 | (-0.086, -0.009) | -0.010 | (-0.038, 0.017) |
| Never married | -0.070 | (-0.130, -0.010) | -0.023 | (-0.077, 0.032) |
| **Wealth (lowest wealth quintile ref category)** |  |  |  |  |
| 2^nd^ | 0.044 | (-0.001, 0.088) | 0.045 | (0.010, 0.081) |
| 3^rd^ | 0.096 | (0.053, 0.139) | 0.122 | (0.087, 0.158) |
| 4^th^ | 0.156 | (0.113, 0.198) | 0.177 | (0.141, 0.213) |
| 5^th^ high wealth | 0.228 | (0.186, 0.271) | 0.240 | (0.202, 0.278) |
| ***Constant*** | *0.839* | *(0.798, 0.88)* | *0.772* | *(0.736, 0.809)* |

*Adjusted for age*

|  | **Men** | | **Women** | |
| --- | --- | --- | --- | --- |
|  | Coef. | 95% CI | Coef. | 95% CI |
| **Marital status (first marriage ref category)** |  |  |  |  |
| Remarried | 0.000 | (-0.039, 0.039) | -0.022 | (-0.066, 0.022) |
| Divorced / separated | -0.063 | (-0.115, -0.011) | -0.048 | (-0.088, -0.008) |
| Widowed | -0.073 | (-0.113, -0.034) | -0.053 | (-0.081, -0.025) |
| Never married | -0.095 | (-0.157, -0.033) | -0.064 | (-0.121, -0.006) |
| **Smoking status (never smoked ref category)** |  |  |  |  |
| Former smoker | -0.041 | (-0.071, -0.012) | -0.016 | (-0.041, 0.008) |
| Current smoker | -0.138 | (-0.188, -0.089) | -0.069 | (-0.111, -0.028) |
| ***Constant*** | *1.012* | *(0.980, 1.044)* | *0.940* | *(0.914, 0.967)* |

*Adjusted for age*

|  | **Men** | | **Women** | |
| --- | --- | --- | --- | --- |
|  | Coef. | 95% CI | Coef. | 95% CI |
| **Marital status (first marriage ref category)** |  |  |  |  |
| Remarried | 0.001 | (-0.035, 0.037) | 0.001 | (-0.040, 0.042) |
| Divorced / separated | -0.050 | (-0.098, -0.002) | -0.051 | (-0.087, -0.015) |
| Widowed | -0.064 | (-0.100, -0.027) | -0.041 | (-0.066, -0.015) |
| Never married | -0.090 | (-0.147, -0.033) | -0.048 | (-0.101, 0.004) |
| **Physical activity (moderate activity ref category)** |  |  |  |  |
| Sedentary | -0.308 | (-0.357, -0.259) | -0.306 | (-0.346, -0.266) |
| Low | -0.156 | (-0.187, -0.125) | -0.170 | (-0.195, -0.146) |
| High | 0.096 | (0.064, 0.128) | 0.054 | (0.021, 0.088) |
| ***Constant*** | *0.987* | *(0.962, 1.013)* | *0.966* | *(0.942, 0.99)* |

*Adjusted for age*

|  | **Men** | | **Women** | |
| --- | --- | --- | --- | --- |
|  | Coef. | 95% CI | Coef. | 95% CI |
| **Marital status (first marriage ref category)** |  |  |  |  |
| Remarried | -0.001 | (-0.040, 0.038) | -0.021 | (-0.065, 0.022) |
| Divorced / separated | -0.073 | (-0.125, -0.022) | -0.057 | (-0.095, -0.018) |
| Widowed | -0.076 | (-0.115, -0.036) | -0.051 | (-0.079, -0.024) |
| Never married | -0.112 | (-0.174, -0.049) | -0.068 | (-0.124, -0.012) |
| **Body Mass Index (BMI<25 ref category)** |  |  |  |  |
| Overweight BMI (25 -29) | -0.004 | (-0.037, 0.029) | -0.043 | (-0.071, -0.015) |
| Obese BMI (30+) | -0.096 | (-0.134, -0.059) | -0.137 | (-0.165, -0.108) |
| ***Constant*** | *1.004* | *(0.968, 1.04)* | *0.987* | *(0.958, 1.017)* |

*Adjusted for age*

|  | **Men** | | **Women** | |
| --- | --- | --- | --- | --- |
|  | Coef. | 95% CI | Coef. | 95% CI |
| **Marital status (first marriage ref category)** |  |  |  |  |
| Remarried | -0.002 | (-0.038, 0.034) | -0.003 | (-0.042, 0.037) |
| Divorced / separated | -0.057 | (-0.106, -0.009) | -0.032 | (-0.067, 0.003) |
| Widowed | -0.075 | (-0.112, -0.037) | -0.038 | (-0.063, -0.013) |
| Never married | -0.114 | (-0.172, -0.056) | -0.056 | (-0.107, -0.005) |
| **Self-rated health (excellent / v. good ref category)** |  |  |  |  |
| Good | -0.075 | (-0.103, -0.046) | -0.111 | (-0.136, -0.086) |
| Fair / poor | -0.251 | (-0.281, -0.22) | -0.296 | (-0.321, -0.27) |
| ***Constant*** | *1.055* | *(1.028, 1.082)* | *1.026* | *(1.002, 1.051)* |

*Adjusted for age*

|  | **Men** | | **Women** | |
| --- | --- | --- | --- | --- |
|  | Coef. | 95% CI | Coef. | 95% CI |
| **Marital status (first marriage ref category)** |  |  |  |  |
| Remarried | 0.004 | (-0.034, 0.042) | -0.019 | (-0.062, 0.023) |
| Divorced / separated | -0.079 | (-0.131, -0.028) | -0.044 | (-0.082, -0.006) |
| Widowed | -0.075 | (-0.114, -0.036) | -0.039 | (-0.066, -0.012) |
| Never married | -0.111 | (-0.172, -0.050) | -0.048 | (-0.103, 0.007) |
| **Chronic health conditions (0 reported conditions ref category)** |  |  |  |  |
| Reported 1 condition | -0.027 | (-0.063, 0.009) | -0.029 | (-0.062, 0.004) |
| Reported 2 conditions | -0.054 | (-0.091, -0.017) | -0.087 | (-0.121, -0.053) |
| Reported 3+ conditions | -0.171 | (-0.210, -0.132) | -0.207 | (-0.242, -0.172) |
| ***Constant*** | *1.017* | *(0.985, 1.049)* | *0.986* | *(0.954, 1.017)* |

*Adjusted for age*

|  | **Men** | | **Women** | |
| --- | --- | --- | --- | --- |
|  | Coef. | 95% CI | Coef. | 95% CI |
| **Marital status (first marriage ref category)** |  |  |  |  |
| Remarried | -0.001 | (-0.039, 0.037) | -0.015 | (-0.059, 0.028) |
| Divorced / separated | -0.069 | (-0.121, -0.018) | -0.041 | (-0.080, -0.002) |
| Widowed | -0.060 | (-0.099, -0.020) | -0.038 | (-0.065, -0.010) |
| Never married | -0.084 | (-0.145, -0.022) | -0.053 | (-0.109, 0.002) |
| **CES-D (CES-D<3 ref category)** |  |  |  |  |
| CES-D≥3 | -0.162 | (-0.198, -0.125) | -0.137 | (-0.162, -0.111) |
| ***Constant*** | *0.991* | *(0.967, 1.016)* | *0.951* | *(0.927, 0.975)* |

*Adjusted for age*

S3L Table: Walking speed and marital status for men and women in HRS regressed on each coefficient individually

|  | Men | | Women | |
| --- | --- | --- | --- | --- |
|  | **Coef.** | **95% CI** | **Coef.** | **95% CI** |
| Marital status (first marriage ref category) |  |  |  |  |
| Remarried | 0.001 | (-0.019, 0.022) | 0.005 | (-0.019, 0.028) |
| Divorced / separated | **-0.062** | **(-0.093, -0.031)** | **-0.066** | **(-0.090, -0.042)** |
| Widowed | **-0.082** | **(-0.110, -0.055)** | **-0.066** | **(-0.083, -0.049)** |
| Never married | **-0.081** | **(-0.140, -0.022)** | **-0.074** | **(-0.122, -0.027)** |
| Age (65-69 ref category) |  |  |  |  |
| 70-79 | **-0.066** | **(-0.086, -0.047)** | **-0.087** | **(-0.104, -0.070)** |
| 80+ | **-0.188** | **(-0.212, -0.163)** | **-0.228** | **(-0.248, -0.208)** |
| *Constant* | *0.873* | *(0.856, 0.891)* | *0.829* | *(0.813, 0.845)* |

|  | Men | | Women | |
| --- | --- | --- | --- | --- |
|  | **Coef.** | **95% CI** | **Coef.** | **95% CI** |
| Marital status (first marriage ref category) |  |  |  |  |
| Remarried | 0.005 | (-0.015, 0.026) | 0.000 | (-0.023, 0.023) |
| Divorced / separated | **-0.037** | **(-0.068, -0.007)** | **-0.039** | **(-0.063, -0.016)** |
| Widowed | **-0.072** | **(-0.099, -0.045)** | **-0.050** | **(-0.067, -0.034)** |
| Never married | **-0.065** | **(-0.122, -0.007)** | -0.038 | (-0.084, 0.008) |
| Ethnicity (white ref category) |  |  |  |  |
| Hispanic | **-0.121** | **(-0.153, -0.089)** | **-0.128** | **(-0.153, -0.102)** |
| Black | **-0.172** | **(-0.199, -0.145)** | **-0.154** | **(-0.174, -0.133)** |
| Other | **-0.038** | **(-0.113, 0.038)** | 0.027 | (-0.037, 0.092) |
| *Constant* | *0.900* | *(0.883, 0.918)* | *0.859* | *(0.843, 0.875)* |

*Adjusted for age*

|  | Men | | Women | |
| --- | --- | --- | --- | --- |
|  | **Coef.** | **95% CI** | **Coef.** | **95% CI** |
| Marital status (first marriage ref category) |  |  |  |  |
| Remarried | 0.000 | (-0.020, 0.021) | 0.004 | (-0.019, 0.028) |
| Divorced / separated | **-0.059** | **(-0.090, -0.028)** | **-0.073** | **(-0.097, -0.049)** |
| Widowed | **-0.080** | **(-0.107, -0.052)** | **-0.066** | **(-0.083, -0.049)** |
| Never married | **-0.080** | **(-0.139, -0.022)** | **-0.078** | **(-0.125, -0.031)** |
| Work status (working ref category) |  |  |  |  |
| Not working | -0.083 | **(-0.105, -0.061)** | **-0.086** | **(-0.107, -0.065)** |
| *Constant* | *0.929* | *(0.906, 0.951)* | *0.895* | *(0.872, 0.917)* |

*Adjusted for age*

|  | Men | | Women | |
| --- | --- | --- | --- | --- |
|  | **Coef.** | **95% CI** | **Coef.** | **95% CI** |
| Marital status (first marriage ref category) |  |  |  |  |
| Remarried | 0.001 | (-0.020, 0.022) | 0.005 | (-0.019, 0.028) |
| Divorced / separated | **-0.062** | **(-0.093, -0.031)** | **-0.066** | **(-0.090, -0.042)** |
| Widowed | **-0.081** | **(-0.108, -0.053)** | **-0.066** | **(-0.083, -0.049)** |
| Never married | -0.056 | (-0.124, 0.013) | **-0.085** | **(-0.138, -0.032)** |
| Parental status (has children ref category) |  |  |  |  |
| No children | -0.034 | (-0.080, 0.012) | 0.016 | (-0.019, 0.051) |
| *Constant* | *0.874* | *(0.856, 0.892)* | *0.829* | *(0.813, 0.844)* |

*Adjusted for age*

|  | Men | | Women | |
| --- | --- | --- | --- | --- |
|  | **Coef.** | **95% CI** | **Coef.** | **95% CI** |
| Marital status (first marriage ref category) |  |  |  |  |
| Remarried | 0.002 | (-0.019, 0.022) | 0.010 | (-0.013, 0.033) |
| Divorced / separated | **-0.055** | **(-0.086, -0.025)** | **-0.063** | **(-0.087, -0.040)** |
| Widowed | **-0.069** | **(-0.096, -0.042)** | **-0.055** | **(-0.071, -0.038)** |
| Never married | **-0.085** | **(-0.143, -0.027)** | **-0.081** | **(-0.128, -0.035)** |
| Education (low ref category) |  |  |  |  |
| Medium | **0.079** | **(0.057, 0.102)** | **0.090** | **(0.073, 0.108)** |
| High | **0.111** | **(0.091, 0.130)** | **0.105** | **(0.085, 0.125)** |
| *Constant* | *0.828* | *(0.809, 0.847)* | *0.788* | *(0.772, 0.805)* |

*Adjusted for age*

|  | Men | | Women | |
| --- | --- | --- | --- | --- |
|  | **Coef.** | **95% CI** | **Coef.** | **95% CI** |
| Marital status (first marriage ref category) |  |  |  |  |
| Remarried | 0.014 | (-0.006, 0.035) | 0.017 | (-0.005, 0.040) |
| Divorced / separated | -0.008 | (-0.039, 0.023) | 0.005 | (-0.019, 0.029) |
| Widowed | **-0.051** | **(-0.078, -0.024)** | -0.015 | (-0.032, 0.002) |
| Never married | -0.046 | (-0.104, 0.012) | -0.020 | (-0.066, 0.026) |
| Wealth (lowest wealth quintile ref category) |  |  |  |  |
| 2^nd^ | **0.052** | **(0.021, 0.082)** | **0.068** | **(0.047, 0.090)** |
| 3^rd^ | **0.098** | **(0.069, 0.127)** | **0.135** | **(0.113, 0.156)** |
| 4^th^ | **0.155** | **(0.126, 0.184)** | **0.179** | **(0.157, 0.201)** |
| 5^th^ high wealth | **0.190** | **(0.161, 0.219)** | **0.200** | **(0.178, 0.223)** |
| *Constant* | *0.750* | *(0.722, 0.779)* | *0.687* | *(0.665, 0.709)* |

*Adjusted for age*

|  | Men | | Women | |
| --- | --- | --- | --- | --- |
|  | **Coef.** | **95% CI** | **Coef.** | **95% CI** |
| Marital status (first marriage ref category) |  |  |  |  |
| Remarried | 0.004 | (-0.017, 0.025) | 0.005 | (-0.019, 0.028) |
| Divorced / separated | **-0.054** | **(-0.085, -0.023)** | **-0.064** | **(-0.089, -0.040)** |
| Widowed | **-0.078** | **(-0.105, -0.051)** | **-0.065** | **(-0.082, -0.048)** |
| Never married | **-0.085** | **(-0.143, -0.026)** | **-0.074** | **(-0.121, -0.027)** |
| Smoking status (never smoked ref category) |  |  |  |  |
| Former smoker | **-0.021** | **(-0.040, -0.002)** | 0.002 | (-0.013, 0.017) |
| Current smoker | **-0.080** | **(-0.110, -0.051)** | -0.013 | (-0.037, 0.011) |
| *Constant* | *0.896* | *(0.875, 0.918)* | *0.830* | *(0.813, 0.847)* |

*Adjusted for age*

|  | Men | | Women | |
| --- | --- | --- | --- | --- |
|  | **Coef.** | **95% CI** | **Coef.** | **95% CI** |
| Marital status (first marriage ref category) |  |  |  |  |
| Remarried | 0.005 | (-0.014, 0.025) | 0.008 | (-0.014, 0.030) |
| Divorced / separated | **-0.050** | **(-0.079, -0.020)** | **-0.054** | **(-0.077, -0.032)** |
| Widowed | **-0.069** | **(-0.095, -0.043)** | **-0.051** | **(-0.067, -0.035)** |
| Never married | **-0.079** | **(-0.135, -0.023)** | **-0.080** | **(-0.124, -0.035)** |
| Physical activity (moderate activity ref category) |  |  |  |  |
| Sedentary | **-0.252** | **(-0.283, -0.220)** | **-0.298** | **(-0.325, -0.270)** |
| Low | **-0.087** | **(-0.108, -0.066)** | **-0.108** | **(-0.123, -0.093)** |
| High | **0.056** | **(0.037, 0.075)** | 0.029 | (0.010, 0.047) |
| *Constant* | *0.878* | *(0.859, 0.897)* | *0.860* | *(0.843, 0.877)* |

*Adjusted for age*

|  | Men | | Women | |
| --- | --- | --- | --- | --- |
|  | **Coef.** | **95% CI** | **Coef.** | **95% CI** |
| Marital status (first marriage ref category) |  |  |  |  |
| Remarried | 0.004 | (-0.017, 0.025) | 0.003 | (-0.020, 0.026) |
| Divorced / separated | **-0.063** | **(-0.094, -0.032)** | **-0.057** | **(-0.081, -0.033)** |
| Widowed | **-0.081** | **(-0.109, -0.054)** | **-0.059** | **(-0.075, -0.042)** |
| Never married | **-0.081** | **(-0.14, -0.022)** | **-0.065** | **(-0.112, -0.019)** |
| Body Mass Index (BMI<25 ref category) |  |  |  |  |
| Overweight BMI (25 -29) | 0.016 | (-0.004, 0.037) | **-0.017** | **(-0.034, -0.001)** |
| Obese BMI (30+) | **-0.033** | **(-0.055, -0.011)** | **-0.108** | **(-0.125, -0.09)** |
| *Constant* | *0.878* | *(0.855, 0.901)* | *0.873* | *(0.855, 0.892)* |

*Adjusted for age*

|  | Men | | Women | |
| --- | --- | --- | --- | --- |
|  | **Coef.** | **95% CI** | **Coef.** | **95% CI** |
| Marital status (first marriage ref category) |  |  |  |  |
| Remarried | 0.007 | (-0.013, 0.027) | 0.012 | (-0.010, 0.034) |
| Divorced / separated | **-0.047** | **(-0.077, -0.017)** | **-0.042** | **(-0.064, -0.019)** |
| Widowed | **-0.071** | **(-0.098, -0.045)** | **-0.044** | **(-0.06, -0.028)** |
| Never married | **-0.068** | **(-0.125, -0.012)** | **-0.059** | **(-0.103, -0.014)** |
| Self-rated health (excellent / v. good ref category) |  |  |  |  |
| Good | **-0.061** | **(-0.080, -0.041)** | **-0.088** | **(-0.103, -0.072)** |
| Fair / poor | **-0.182** | **(-0.202, -0.162)** | **-0.216** | **(-0.233, -0.200)** |
| *Constant* | *0.937* | *(0.917, 0.956)* | *0.900* | *(0.884, 0.917)* |

*Adjusted for age*

|  | Men | | Women | |
| --- | --- | --- | --- | --- |
|  | **Coef.** | **95% CI** | **Coef.** | **95% CI** |
| Marital status (first marriage ref category) |  |  |  |  |
| Remarried | 0.005 | (-0.016, 0.025) | 0.012 | (-0.011, 0.035) |
| Divorced / separated | **-0.063** | **(-0.093, -0.032)** | **-0.050** | **(-0.073, -0.026)** |
| Widowed | **-0.078** | **(-0.105, -0.051)** | **-0.053** | **(-0.070, -0.037)** |
| Never married | **-0.082** | **(-0.140, -0.024)** | **-0.068** | **(-0.114, -0.022)** |
| Chronic health conditions (0 reported conditions (ref category) |  |  |  |  |
| Reported 1 condition | **-0.020** | **(-0.054, 0.014)** | **-0.032** | **(-0.063, -0.001)** |
| Reported 2 conditions | **-0.053** | **(-0.085, -0.020)** | **-0.078** | **(-0.108, -0.048)** |
| Reported 3+ conditions | **-0.122** | **(-0.153, -0.090)** | **-0.163** | **(-0.192, -0.133)** |
| *Constant* | *0.934* | *(0.903, 0.965)* | *0.911* | *(0.881, 0.94)* |

*Adjusted for age*

|  | Men | | Women | |
| --- | --- | --- | --- | --- |
|  | **Coef.** | **95% CI** | **Coef.** | **95% CI** |
| Marital status (first marriage ref category) |  |  |  |  |
| Remarried | 0.004 | (-0.016, 0.025) | 0.006 | (-0.017, 0.029) |
| Divorced / separated | **-0.047** | **(-0.078, -0.016)** | **-0.055** | **(-0.079, -0.032)** |
| Widowed | **-0.063** | **(-0.090, -0.035)** | **-0.053** | **(-0.07, -0.037)** |
| Never married | **-0.068** | **(-0.126, -0.009)** | **-0.069** | **(-0.115, -0.023)** |
| CES-D (CES-D<3 ref category) |  |  |  |  |
| CES-D≥3 | **-0.115** | **(-0.139, -0.092)** | **-0.124** | **(-0.140, -0.108)** |
| *Constant* | *0.886* | *(0.868, 0.904)* | *0.848* | *(0.833, 0.864)* |

*Adjusted for age*
